# The irreplaceable role of sleep in building long-term memories: offline integration of free cortical units and its possible implication in autism

**DOI:** 10.3389/fnhum.2026.1777687

**Published:** 2026-08-14

**Authors:** Dominique G. Béroule

**Affiliations:** LISN (CNRS), Campus Universitaire, Orsay, France

**Keywords:** autism spectrum disorder, axonal sprouting, cortical columns, guided propagation network, ionic signalling, thalamocortical circuits, time-place coding, epigenetic regulation

## Abstract

The part played by synaptic plasticity in natural learning can be reassessed owing to the discovery of dormant neurons in the cortical layer-2 of large-brained mammals. Here, the recruitment of *cortical columns* that contain these quiescent cells is argued to underlie the lifelong building of long-term memories, albeit only during periods when cortical regions are segregated, while perception, cognition and motor skills are at their lowest level. As records of online experience are replayed by the hippocampus, about half of every sleep cycle would harbour the relevant *neurites* outgrowth between in-use and free cortical columns. This translation of labile traces into steady imprints is simulated on computer, in an attempt to link experimental findings to specific functions and possibly generate predictions. Rapid eyes movement observed at the end of every sleep cycle is notably interpreted as a side effect of the necessary priming of peripheral effectors whereby cortical replay can induce activation in the hippocampus and thus allow new connections between paths that are thus concurrently activated. Architectural and functional data highlight a topological matching between key components of the underlying model and those of *thalamocortical* structures. Several factors are considered for the experience-driven development of these structures, including slow-wave activity whereby the guided growth of axons may occur from layer-2 of their host column towards the layer-4 of free columns, possibly steered in parallel by distinct calcium waves. In addition to this main prediction, this theory supports the central role of sleep in the development of autistic symptoms. Rooted in an epigenetic downregulation of the MAOA brain enzyme, the resulting offline over-activity would entail aberrant branching between *cortical columns*. Whereas most features of the condition have already been interpreted through this systemic approach, the evolution of autism prevalence across four human generations is derived here from the masking effects of genetic variants and X chromosome inactivation, while a host of risk factors is liable to promote MAOA. Accordingly, pregnancy should be prevented from excess MAOA activity by being subjected to prior blood testing, which may lead to the individualized prescription of MAOA inhibitors.

## 1 Introduction

*“The cerebral cortex resembles a garden populated by innumerable trees, the pyramidal cells, which, thanks to intelligent cultivation, can multiply their branches, sink their roots further and further, and produce flowers and fruits that grow more exquisite every day.”* (Ramón y Cajal, 1894)

Bird brains have long provided a fertile field of investigation for neuroscientists, notably allowing Ramon y Cajal to uncover microscopic gaps between nerve cells, later called *synapses*. That the variable content of synaptic gaps could mediate the transmission of neural signalling opened a highway to theories of memory formation, whereby the pieces of a near-solved puzzle could in the adult brain be adjusted and refined under learning pressure. Owing to his numerous drawings of the tree-like growth of bird brain cells, Cajal also anticipated the *prenatal* birth of new neurons (i.e.: *neurogenesis*), although he observed morphological differences between the same type of neurons in human foetuses and newborns (Gil et al., 2014). He further assumed the establishment of new pathways at the basis of learning thanks to the targeted growth and branching of neural arborization, a foresighted view proposed as early as the dawn of the 20th century (Rozo et al., 2024). Had it been less dominated by the doctrine that no new neuron integrates the brain after birth, later research on the physiological substrate of memory might have enabled new “puzzle pieces” to join in the lifelong updating of memories. But since Cajal, scientific contributions (e.g.: *Pavlov conditioning* of sensorimotor pathways) rather imposed the selective consolidation of synapses, especially after its linking with molecular mechanisms known as *Long-Term Potentiation* (for a review: Abraham et al., 2024).

### 1.1 Adult neurogenesis and the brain size

In the 80’s, a scientific breakthrough was achieved through the discovery of *adult neurogenesis* in songbirds, notwithstanding the bias of their relatively small brain. Migrating from *neurogenic niches* where they proliferate, newborn neurons were found to differentiate in several areas of the bird adult brain: hippocampus (HC), striatum and the *high vocal centre* responsible for the control of song production (Nottebohm, 1989). At that time, the discovery that a complex sensorimotor behaviour such as song learning builds up on new neurons could suggest a similar phenomenon in human neocortex. However, approximating the age of neurons -whose DNA is marked by the exponential decrease of *Carbon 14* since 1963—put a halt to this expectation by providing evidence that no *occipital* cortical neuron is created after birth in humans (Spalding et al., 2005): “whereas non-neuronal cells turn over, neurons in the human cerebral neocortex are not generated in adulthood at detectable levels but are generated perinatally” (Bhardwaj et al., 2006). Adult neurogenesis was dismissed, exclusive of its significant contribution to both HC and striatum, especially in human (Ernst and Frisén, 2015). Since then, other birth-dating analyses confirmed a verdict at odds with the dynamic incorporation of newborn neurons into the postnatal neocortex (Llorens-Martin and Trejo, 2011), but into the olfactory bulb (Dejou et al., 2024), whereas immature inhibitory interneurons may continue to migrate into the human frontal lobe at least five months after birth (Paredes et al., 2016).

The data above is particularly challenging for the biological credibility of memory models wherein the sporadic and autonomous acquisition of knowledge is committed to the lifelong enrolment of new functional units (Béroule, 1988), a concept that was elusive until the discovery of potential candidates for this recruitment. Instead of newborn cells migrating from short-range subcortical niches (e.g.: in bird, and rodent), the adult cortex of large-brained mammals would more effectively host a pool of *dormant cells*. Not actually new, but immature and in a pending standby mode, neuronal precursors have indeed been found in the adult neocortex (Bonfanti and Nacher, 2012). Among the full set of embryonic stem cells, some apparently head for cortical (CX) regions without right away serving the brain development. Years or even decades later, these perennial adolescent neurons stuck in a stand-by state could resume their growth by adopting traits that meet architectural needs at that time. In agreement with this view, cells that express the *doublecortin* (DCX+) marker of immaturity have been identified in adult primates (Zhang et al., 2009) as a reservoir of “aspiring neurons.” These potential add-on units are located in layer 2 of mammal cortices, with more than two million DCX+ cells estimated to exist in chimpanzee’s cortex, one hundred in bats and none in mice (La Rosa et al., 2020). During their evolution, in parallel with neurogenic niches, certain brains would thus reserve CX neural precursors issued from prenatal migration. In line with the different types of plasticity noted by Bonfanti et al. (2024), small brained species implying relatively short distances for these *neural stem cells* (NSC) to travel would accommodate migration in order to complete neural circuits that support new behaviours (e.g.: production of a novel song at the mating season). NSCs would in small brain migrate postnatally before eventually dividing up towards a cortical target. Otherwise, learning would better harness free quiescent cells already *in-situ*. In other words, the largest brains may implement quiescent neurons already on-the-spot. From this new perspective, the question arises as to whether dormant cells could throughout life be awakened and effectively integrated on learning demand to the existing neural network.

### 1.2 The brain runs blind and motionless across sleep

Sleep is mostly thought of as a substantial period of inaction, a privileged resting-time for regaining strength. On this background, specific processes ensure a well-functioning brain by cleaning metabolic end-products of wakefulness, and restoring key molecules (e.g.: *growth hormone*). Improved well-being could be observed in individuals suffering from chronic pain, indicating that worse sleep quality was associated with lower scores on a “Satisfaction with life” scale (Seweryn et al., 2023). At first glance, both perceptual withdrawing and absence of movement are meant to replenish sensorimotor systems. However, the latter are still centrally controlled by the brain, which consumes only 20% less energy during slow wave sleep (SWS) than over its “online” state of alertness and across paradoxical sleep (PS). The “offline” and motionless behaviour at issue can also be assumed to conceal work undertaken by the brain under more appropriate conditions than while “online.” According to studies which evidence the role of sleep in memory, the decreased sensitivity of the brain to sensory events would indeed prevent the consolidation of memory traces from being impeded by external stimuli (Barnes and Wilson, 2014). Other arguments can be put forward to resolve this contradiction between an everlasting active brain and the cyclic inaction of its host body, notably through modelling trials.

**Figure 1:**
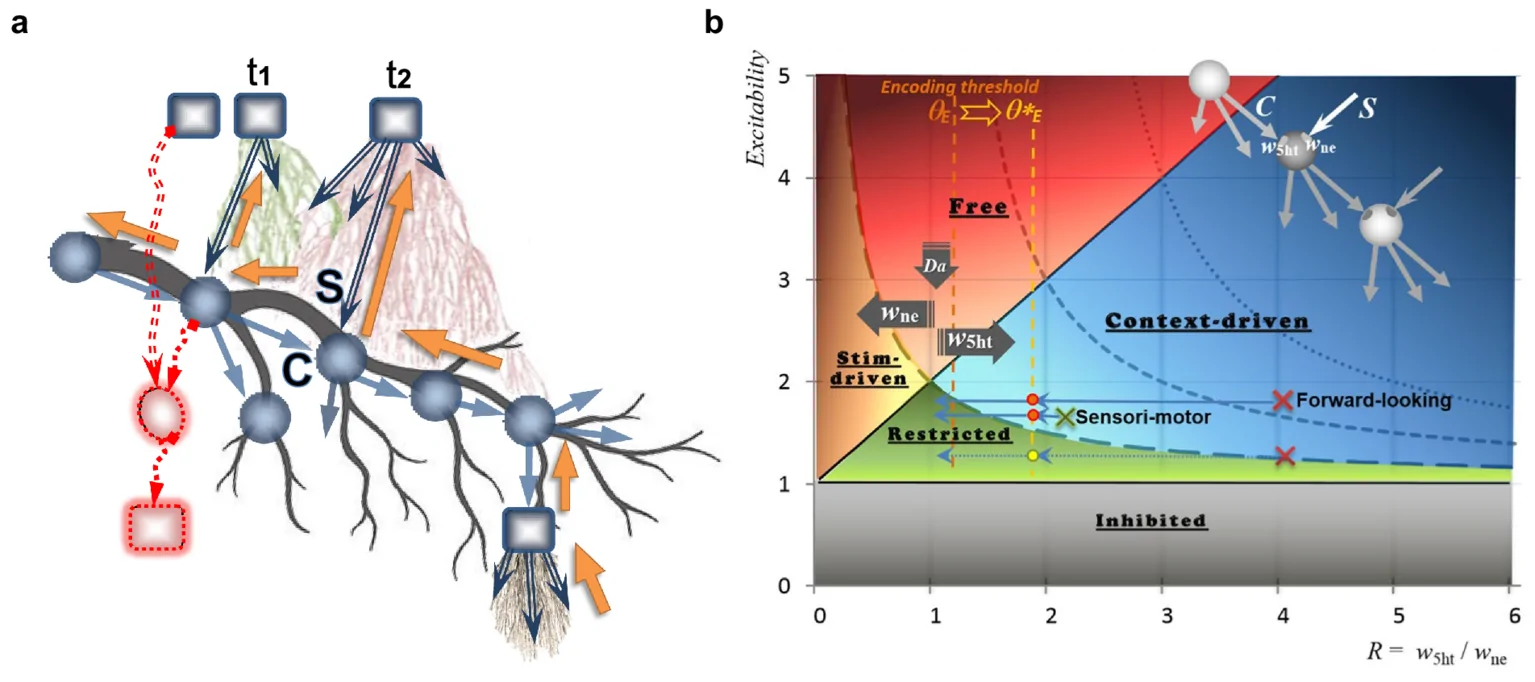
Guided propagation (GP) of activity along the branches of a tree-like structure: a generic process for the recognition, generation and learning of sequences of instantaneous events (stimuli). **(a)** A tree-like GP module propagates its activity (blue-grey rightwards arrows) which is guided in the course of time (t_1_, t_2_, …) by stimuli (downwards empty arrows) and backward modulating signals (orange leftwards and upwards arrows). Each functional unit (round symbol) intersects a tree-branch that conveys contextual information (C) and a shrub-branch which spreads a stimulus (S). Activity issued from the root (left-most unit) can be guided towards the tip of a branch/path (e.g.: shrub plotted at the bottom) by a sequence of stimuli, and can guide in turn the inner activation of a deeper module. Upstream modulating signals may facilitate or hinder the recognition of a sequence of events, recall/replay the latter without external stimuli, or generate a sensorimotor pattern paced by proprioceptive stimuli. A GP module grows through sporadic branching (in dotted red lines) when stimuli occur that have not been expected by the C short-range contextual flow. **(b)** Excerpt from Béroule (2018). Basic processing modes of a GP unit (a memory path of 3 units is plotted in the upper right corner) as a function of its *Excitability* (response threshold) and ratio *R* between weights of its contextual (C) and Stimulus (S) inputs. In the basic “restricted propagation” mode, both inputs are required for the units’ response thresholds to be reached, thus implementing strict “coincidence detection” (yellow dot). A low Excitability (e.g.: red cross with (4, 1.2) coordinates at the border between the green and blue areas) ensures propagation to be “restricted” and avoids the context-driven zone (e.g.: “Forward-looking” cross in the blue area) which is not suitable for the dynamic integration of new units. Both “Free” and “Stimulus-driven” areas are always to be avoided, for they may entail irrelevant (out of context) responses. Da, ne and 5ht refer to GP variables akin to Dopamine, Noradrenaline and Serotonin neurotransmitters.

In the so-called Guided Propagation (GP) model, sequences of discrete events (shortened to “trajectories” no matter their aim, either perceptive or motor) are stored in the form of tree-like branches. New branches are grown through *learning by differentiation* when unexpected events occur, and represent several trajectories within a GP module (Figure 1a). Central for a model that relies on the replay of trajectories, the activation of a given branch (or “memory path”) can be steered by two means. (1) On the one hand, as known stimuli occur in chronological order at the module input, they guide the spontaneous activity stemming from the tree-root onwards a specific branch. (2) On the other hand, a depolarizing wave sent by other modules may spread backwards a path in order to facilitate/prime or induce the full activation of the targeted path and its input. In a path intended for generating a motor sequence, this wave pulls the spontaneous activity, activates effectors that feedback proprioceptive stimuli whereby the ongoing trajectory is paced. The same pull/push mechanism is at work in active perception involving proprioception from peripheral systems. Consistently with the *mirror neuron* system (Rajmohan and Mohandas, 2007), GP memory paths can therefore both perceive and generate their associated series of input/output events, making them potentially instrumental in modelling sensorimotor *learning by imitation*.

The foregoing enables trajectories to be replayed and possibly stored elsewhere, preferably without being disturbed by several types of noise. Because some sensors are meant to dynamically report on self-movement and body position (*proprioception*), motion should be avoided. Similarly, outside (*exteroceptive*) stimuli are not welcome, for they would interfere with the replay of former records to be assigned “private” memory paths. On top of that, the completion of existing paths can be disrupted by modulating flows emitted by other modules, causing the path activity to spread ahead of stimuli in the “context-driven propagation” mode (Figure 1b), with differentiation unfolding in a disorderly and exaggerated growth (Béroule, 2018). All in all, there may be a chance that some peculiar visions of Ramon y Cajal meet reality, insofar as constraints are fulfilled. For cortical neurons to actually “multiply their branches” through “*intelligent cultivation*,” one condition could be the cyclic intrusion of periods wherein the brain is disengaged from both proprioceptive and exteroceptive perceptions, as well as from signalling between regions, thus issuing their *segregation*.

Data on sleep and HC signalling (Béroule, in press) lends support to the above view. Accordingly, generic paths could online be instantly locked to distinct pulse signals coding for as many trajectories to be replayed offline. During this later period, unless CX is not ready to learn new material, the successful translation into persistent memories of labile records can relieve HC from the storage of these very records.

Here, computer simulations show how and under which conditions HC and CX may interact so as to run the offline transformation of memories. Functional and architectural similarities between GP modules and subsets of *cortical columns* are then emphasized, in support of the study of psychiatric conditions with the GP model. In that respect, a previous investigation (Béroule, 2018) that linked up autism spectrum disorder (ASD) with sleep will be resumed, and completed by a focus on ASD evolution over human generations.

## 2 Interplay of complementary codes across online/offline cycles

To avoid potential confusions between the biological complexity and an instance of simplistic model, the subset of HP and CX functions modelled here will in the following be named *Hpc* and *Ctx*, respectively. Owing to their common GP substrate and processing method, *Hpc* and *Ctx* are predisposed to exchange information through steps to be delineated.

As previously stressed, the brain is far from inactive after having gradually switched from arousal and vigilance to the stationary unconsciousness of sleep. Freed from the control of behaviour encompassing anticipation and action, cortical regions would then be ready to acquire knowledge of situations previously recorded by the hippocampus, among which some are already known. This hypothesis raises the issue of whether—and how—the cortical hardware could support such sporadic learning performed without external supervision.

### 2.1 Signalling between *Hpc*/HC and *Ctx*/cortical areas

Together with root-units that initiate the inner flows of activity, peripheral sensors/effectors belong to the pre-built frame of any GP architecture, and are already cross-connected to ensure initial *Hpc-Ctx* communication. Therefore, each peripheral ‘ground floor’ unit is originally linked to its homonym at the entrances of other channels. Pre-existing as well, root-units initiate the inner contextual streams in every module. Memory formation can be derived from this backbone architecture (see video screenshots taken during computer sessions: Béroule, 2023).

Although different in nature, the coordinated activity of *Hpc* and *Ctx* memory paths recalls the locked timing of HC ripples and cortical signals (Jiang et al., 2017, 2019): Under the assumption that the duration of fast oscillations (i.e.: spindles) matches the fast environment-free replay of sequences, the depolarization of ‘anterior HC’ ripples indeed compares with the series of GP thresholds pulse-like drops that elicit the generation of trajectories to be produced (Béroule, in press). With respect to the close timing of the two channels that are represented here, the early increase in *Ctx* spontaneous activity is monitored to simulate the “*Ctx* audience” attention to *Hpc* channel performance. GP running simulations therefore show that the first active channel neither necessarily indicates the origin of “information transfer” nor enhances a prominent/leading role. Unlike the direct transfer of information usually assumed from HC to targeted CX areas (Kitamura et al., 2017), *Hpc* sparse activity is rehearsed and widely broadcasted, regardless of which *Ctx* channels are sensitive to a particular production.

Communication between *Hpc* and *Ctx* channels must be prepared beforehand. In fact, their ground floor composed of lower-level events behaves like a mirror that projects the trajectories produced by one side towards the other. Given that GP production is based on threshold drops (facilitating/depolarizing signals), the latter should meet subthreshold activation (or *priming*) in its own channel for issuing responses. Along the *Hpc* → *Ctx* pathway, any *Hpc* production by “storey −1” first propagates upwards towards the ground-floor and then downstream of the *Ctx* channel, but only once the ground-floor has issued activatory guiding feedback that witnesses the effectiveness of each production step. Within the motionless offline period, the required feedback can result from the prior priming of all ground-floor units, just below their response thresholds. Along the opposite direction, *Ctx* can also spread “backwards facilitation” upwards peripheral organs of active perception. This mechanism may stimulate movements in sensors when *Ctx* generates sensorimotor patterns intended for *Hpc*, whereas *Hpc* replay does not cause the same facilitation pattern upwards the *Ctx* ground-floor. This distinctive interplay between facilitating and activating signals in *Hpc* and *Ctx* generates a new hypothesis regarding the physiological origin of rapid eyes movement (REM) that occur over PS. Projected into the biological context, corresponding mechanisms should logically not only concern eyes effectors but also active components of hearing such as *outer hair cells* (Brownell, 1990; Cooper and Guinan, 2006) and even the *basilar membrane* of the cochlea.

**Figure 2:**
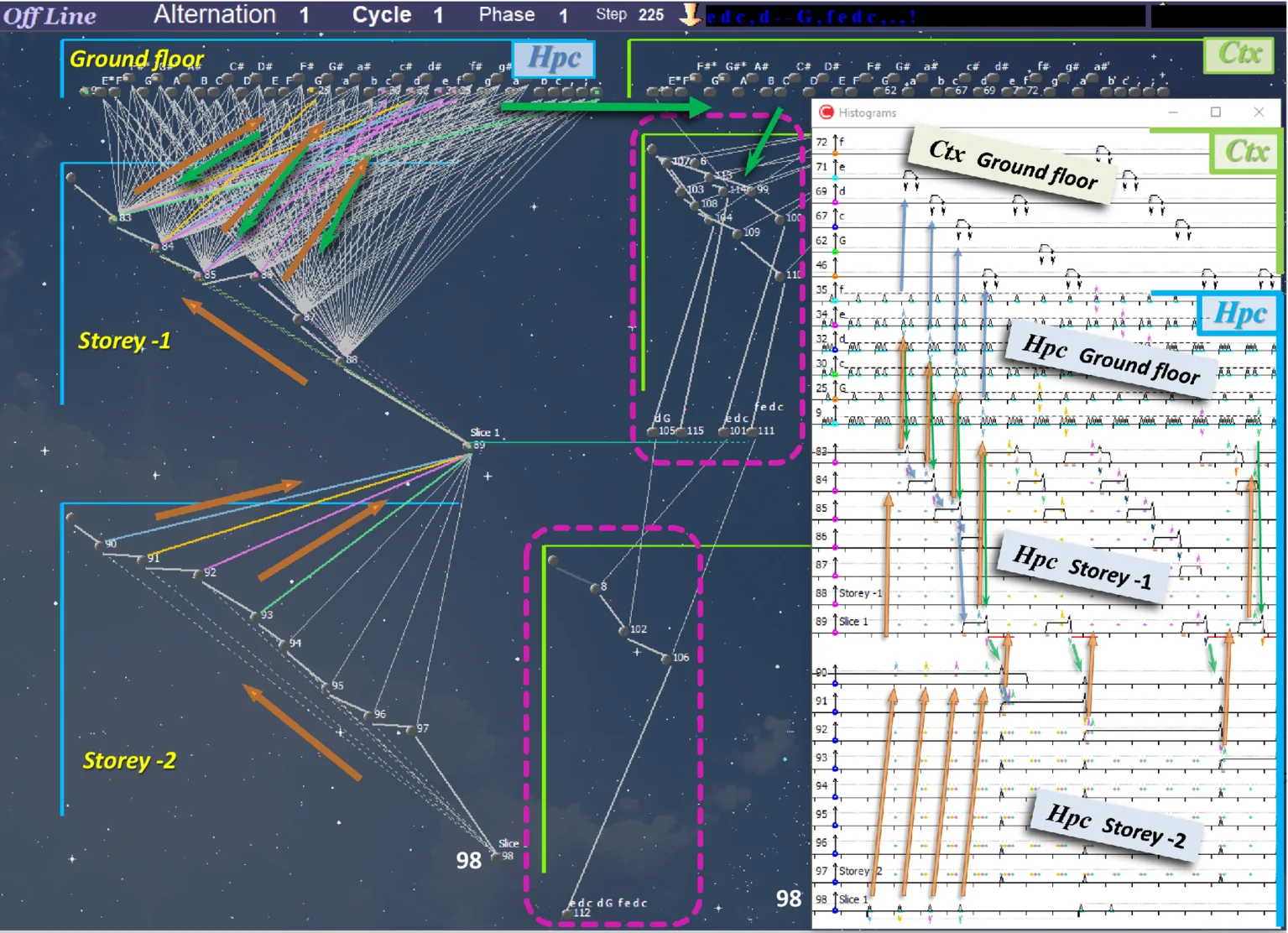
GP network after the building of private *Ctx* memory paths from *Hpc* replay. Surrounded here by purple dotted frames, new *Ctx* paths result from the repetitive generation of trajectories previously recorded by the *Hpc* generic paths of two storeys (chained cells to the left). As in figures to follow, a screen shot of the software output has been completed with informative labels, arrows and frames. In *Ctx* histograms displayed in the pop-up window to the right, sub-threshold bursts are repeatedly generated at 4 phases previously assigned online. Then, facilitation (orange arrows) is initiated at the bottom of Storey -2 (node 98), triggering an ascending wave of 4 threshold drops meant to generate 4 motives in storey -1. A few of the ground-floor primed units eventually respond, thus bringing about relevant tonic signals at the *Ctx* channel input (‘*Ctx* ground floor’ histograms to the top). Among all *Hpc* productions, only those not anticipated by *Ctx* can trigger and then accompany the sprouting of new *Ctx* paths. Once completed, the latter are eventually connected to their *Hpc* generic partner (blue horizontal lines) through *Ctx* replay.

### 2.2 Online *Hpc* encoding and *Ctx* running

In computer simulations of online encoding, a turnover process monitors the assignment of *Hpc* slices and phases to trajectories. Each slice is filled with as many trajectories as available phases, before handing over the next slice which in turn integrates the same set of phases for coding future trajectories. Processed by the slice which is currently active, an incoming trajectory is therefore tentatively assigned the next free phase.

On-line, the *Ctx* and *Hpc* channels are concurrently stimulated by the same input. While *Hpc* is recording significant events on the fly, *Ctx* is busy matching the same events against its inner references for guiding behaviour. The output level (tonic signal) of a *Ctx* path can provide a matching-score (Westerlund et al., 1996) whose lowest values highlight trajectories that deserve being recorded by *Hpc*. On condition that crosslinks are created between *Ctx* and *Hpc* paths every time a *Ctx* path has finished growing, *Hpc* can be informed online about the convenience of its current recording. Given that a new input may only differ from a known pattern by a final stimulus, this is only at the end of a trajectory that the ongoing *Hpc* phase assignment can be acknowledged by the *Ctx* channel, which is straightforward when none of its existing paths has concurrently been activated. In contrast, the full activation of a *Ctx* path witnesses an already existing permanent storage; the current *Hpc* phase assignment is dismissed and thus made available for tentatively binding elements of a forthcoming new trajectory.

Possible hippocortical interaction n°1: Online, *Ctx* = > *Hpc*

*Ctx* prevents *Hpc* from encoding already known trajectories

If available, a new slice is brought into play whenever all phases have been used. By drawing on the same pool of *Nb_pha* distinct signals, every *Hpc* slice (generic path plus its associated input/output) can serve the later acquisition of as many new memories by *Ctx*. Thanks to separate lectures then given by *Nb_HpcSli “*tutoring slices,” *Ctx* will offline be stimulated by *Nb_pha* x *Nb_HpcSli* new trajectories in total.

### 2.3 Offline *Hpc* replay and *Ctx* encoding

Offline, the aforementioned segregation of modules allows the possible outgrowth of *Ctx* memory paths. For this purpose, two parameters that determine the units’ responsiveness are set in the “restricted propagation” area (Figure 1b), which implements segregation by avoiding modules to spread facilitation/inhibition elsewhere in the way emotional modules guide decision-making (Béroule and Gisquet-Verrier, 2016). Thus withdrawn from the influence of other modules, *Ctx* memory paths are ready to properly grow under the only stimuli issued from *Hpc* replay (Figure 2).

**Figure 3:**
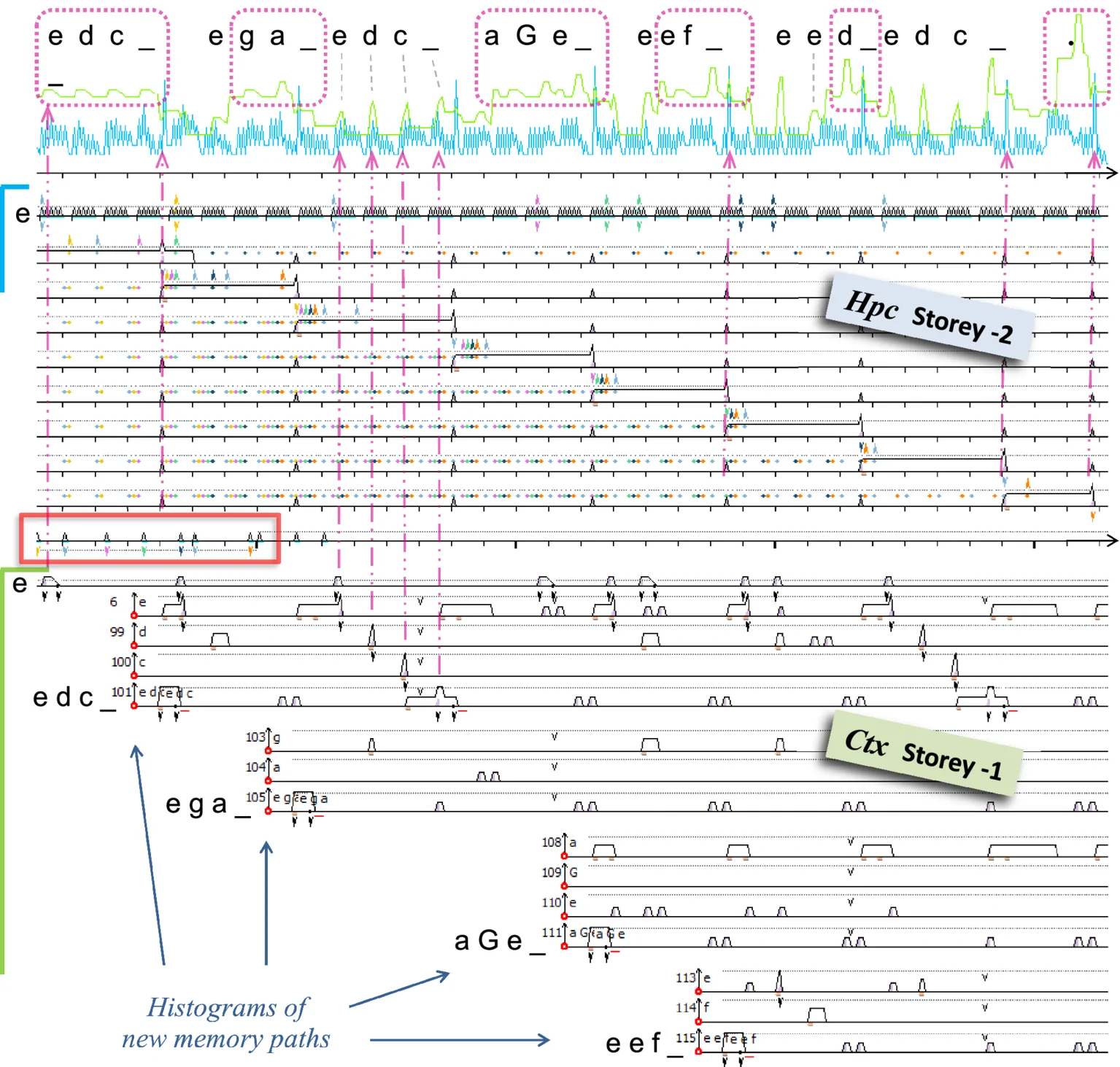
Mapping of the global activities of phasic (*Hpc*) and tonic (*Ctx*) channels (above the upper time axis) onto their local signals components. Letters displayed at the top of the figure stand for notes of the “Let it be” melody (The Beatles, 1970) that are rehearsed by *Hpc*. The blue spiking curve shows the global *Hpc* high-rate phasic activity tied to the replay in progress. The green curve at the top shows *Ctx* global activity the level of which depends on the predictability of the ongoing trajectory: Unexpected notes are shown surrounded by a purple frame. When a musical motive is not already represented in the peripheral storey -1 of *Ctx*, the wave intensity reflects the local over-activation of a path under construction, whereas more discrete bursts accompany the identification of a known pattern (i.e., first occurrence of ‘e d c’ as compared to following ones). Besides, the global tonic activity tends to increase with the incorporation of add-on units into *Ctx*. Owing to the software parameters used in this figure, free units can instantly be connected, allowing a new private path to be built over a single replay of *Hpc* pulses (framed here by a superimposed red rectangle). Although not consistent with the neural growth rate, this one-shot learning enables viewing the onset of histograms that are automatically displayed on new path completion (five diagrams at the bottom).

*Hpc* deep-storey (level −2) output units express subthreshold spikes at the characteristic phases they have previously been assigned online, for being ready to generate relevant trajectories through selective depolarization. Waves of phasic facilitation can repeatedly flow upstream a *Hpc* deep generic-path, from storey −2 towards the ground floor, in order to dispatch trajectories “to whom it may concern” in *Ctx* (Figure 3). As previously stated, the *Hpc* ground floor must also concurrently generate all its phasic signals at a sub-threshold level so as to prepare the upcoming replay. Analogous to depolarization, a wave of response thresholds ‘negative pulses’ at a given phase can then produce any sequence of events previously bound to this very phase. Meanwhile, the *Ctx* channel triggers spontaneous flows to be guided by stimuli issued from *Hpc*. Thanks to the cross-connections between the *Hpc* and *Ctx* ground-floor units, every offline *Hpc* production sounds for *Ctx* as if coming from the outside world, albeit at the highest possible rate determined by immediate feedbacks from the *Hpc* ground-floor.

Possible hippo-cortical interaction n°2: Offline, *Hpc* = > *Ctx*

*Hpc* is replaying trajectories to be durably stored in *Ctx*

**Figure 4:**
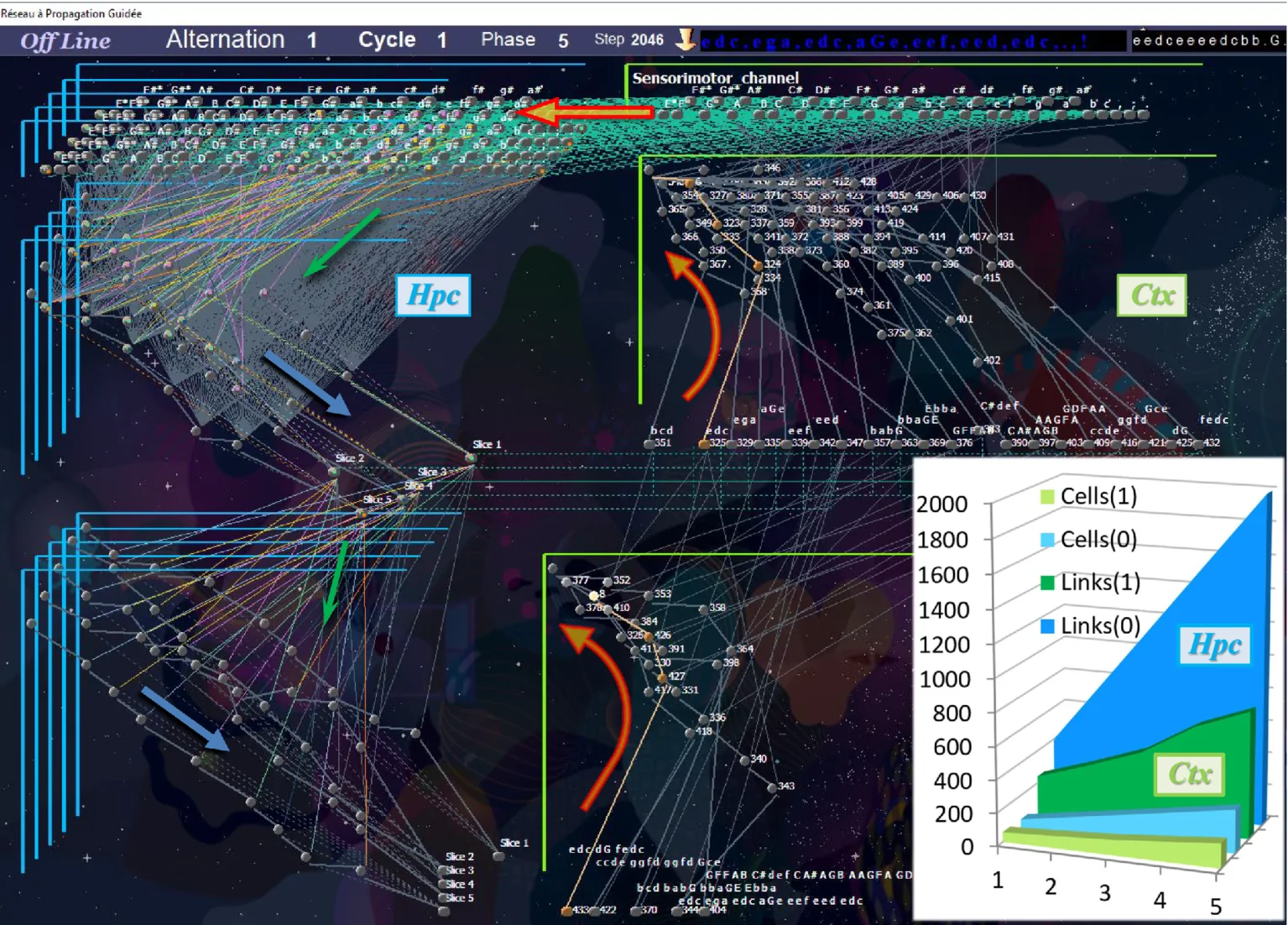
At the end of an offline cycle when deep *Ctx* paths are replayed, quantitative comparison between the content of *Hpc* and *Ctx* that have been fed with the same input. On the left-hand side, 5 melodies are stored within 5 *Hpc* slices, respectively. On the right-hand side, two *Ctx* paths (in orange) are currently busy replaying one of these compound patterns (“Give peace a chance”, labelled ‘edc dG fedc’), just after the completion of its *Ctx* path in Storey 2. The bottom-right pop-up window displays the number of links and functional units (“cells”) that have been recruited in *Hpc* (in blue) and *Ctx* (in green) storeys, respectively. *Hpc* generic paths have used approximately twice as many resources in terms of cells and links than the *Ctx*-specific paths. However, the same generic paths will be used again with recycled phases to keep track of new trajectories to occur afterwards.

**Figure 5:**
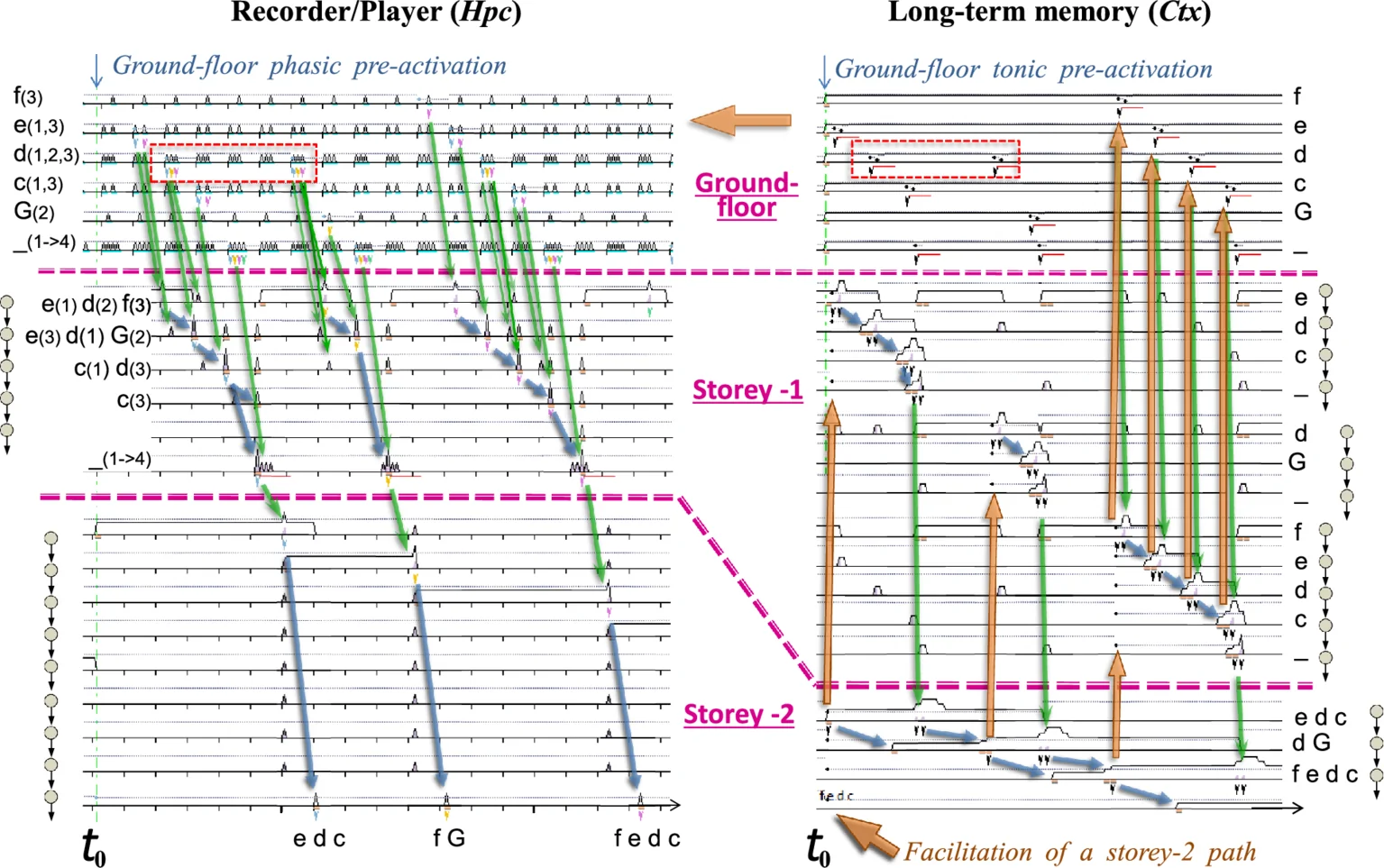
Offline replay of a deep *Ctx* path (in storey -2) and resulting activation of a deep *Hpc* generic path. At time *t*_0_, root-units of both *Ctx* and *Hpc* channels are primed ahead of the tonic facilitation (upwards orange arrows) of the path labelled “edc dG fedc” and plotted at the bottom-right. Relevant threshold drops drive the Storey -1 spontaneous flow along paths of musical motives (edc, dG, fedc) that together form the “Give peace a chance” melody. Once reached the *Ctx* ground floor (top of the figure), threshold ‘square drops’ are produced by *Ctx* effectors towards their *Hpc* correspondents where they possibly coincide with phasic bursts. To the left-hand side, the labels associated with generic path cells specify within brackets the stimuli (notes) and phases to which they have been tuned online, namely one phase per trajectory (musical motive, here). The resulting time–space pattern of stimuli spreads down the *Hpc* storeys in a way comparable to the actual occurrence of the same stimuli stemming from the outside world, albeit with extra ambiguity partly due to the tonic/non-specific *Ctx* signals. However, despite the systematic activation of every *Hpc* phase associated with a given stimulus (“d”, framed by red dotted rectangles), the contextual pulse signals conveyed by the *Hpc* Storey -1 can enhance the relevant phase through coincidence-detection.

A reservoir of *in-situ Ctx* units serves the building of new paths over *Hpc* replay (Figure 2). Since the growth rate lags behind *Hpc* productions, a given *Ctx* path can only increase step-by-step at every *Hpc* replay, update its contextual activation and resume growth, and so on (see video: Béroule, 2023). From the beginning: spontaneous activity is launched after the full reset of *Ctx*. The first time an unexpected event is generated by *Hpc*, *Ctx* responds by sprouting a new branch from the spot reached by the inner flow along an existing path/branch of a module tree (i.e.: learning by Differentiation). Next occurrences of the same trajectory, whatever the time-delay between them (sporadic learning), will reactivate the relevant path under construction so as to reinstate the suitable context for “axonogenesis” to resume. Once fully built, new *Ctx* paths are played in turn (Figures 4, 5), which corresponds to the paradoxical sleep stage initiated by the facilitation of deep storeys and cross-channel circuits (Supplementary Figure S1). Each *Ctx* production makes *Hpc* respond slightly later, inducing a feedback connection between a new *Ctx* path end and its *Hpc* generic counterpart (horizontal dotted blue lines in Figures 2, 4). These cross-circuits enable the “Hippo-cortical interaction n°1” during online phases.

Possible hippocortical interaction n°3: Offline, *Ctx* = > *Hpc*

*A Hpc* generic path is notified that one of its trajectories has been learnt by *Ctx*

Even though the final outcome compares with a transfer of information, online records are rather rehearsed offline and widely broadcasted towards several potential *Ctx* “listeners,” each being specialized in one aspect of the *Hpc* performance. Once completed, the storage of a given trajectory in *Ctx* may relieve *Hpc* from its “buffering” role. Regarding neurobiological data, for hippocampus traces to be fully translated into cortical ones, the observation of engram cells showed that a few weeks are required for their functional, structural and physiological maturation; the hippocampus action stops afterwards (Li et al., 2017).

**Figure 6:**
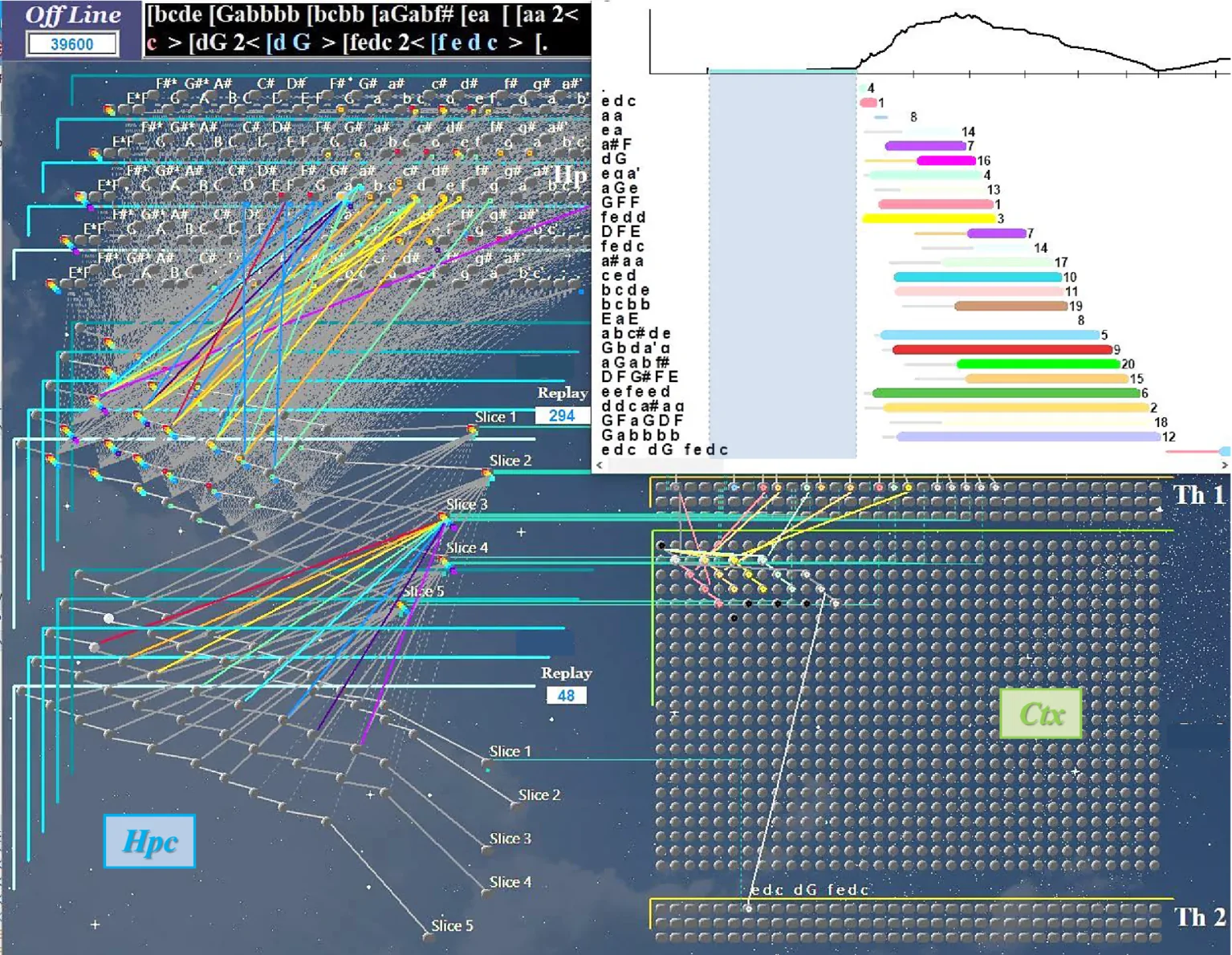
Offline growth of parallel *Ctx* paths steered by distinct waves. The matrix of *Ctx* units to the right-hand side represents a pool of canonical cortical circuits (little round cells). The banks of input/output cells at the interface of the *Ctx* storeys are labelled Th1 and Th2, respectively, where “Th” stands for “Thalamus”. Here, the deepest *Hpc* storey slices alternately broadcast 5 melodies previously recorded online, that combine 20 musical motives recorded in the more peripheral module. The resulting simultaneous extension of 20 paths in the Storey −1 of *Ctx* (hidden here by a pop-up window) relies on a number of carrier waves (identified by distinct colors and numbers) visualized by the line graph at the top-right, over 6 replay cycles (6 successive intervals along the time axis). This screenshot was taken while the 2nd storey was in turn busy encoding full compound trajectories replayed by *Hpc*. Among the 5 relevant paths that are currently built in storey −2, “Give peace a chance” has just been finished and therefore has received a label made from concatenating its “component units” labels. As learning proceeds together with the recycling of involved waves, every completed path connects to its associated *Hpc* generic path, which can be released from its ongoing phasic code while the *Ctx* path is freed from its carrier wave.

**Figure 7:**
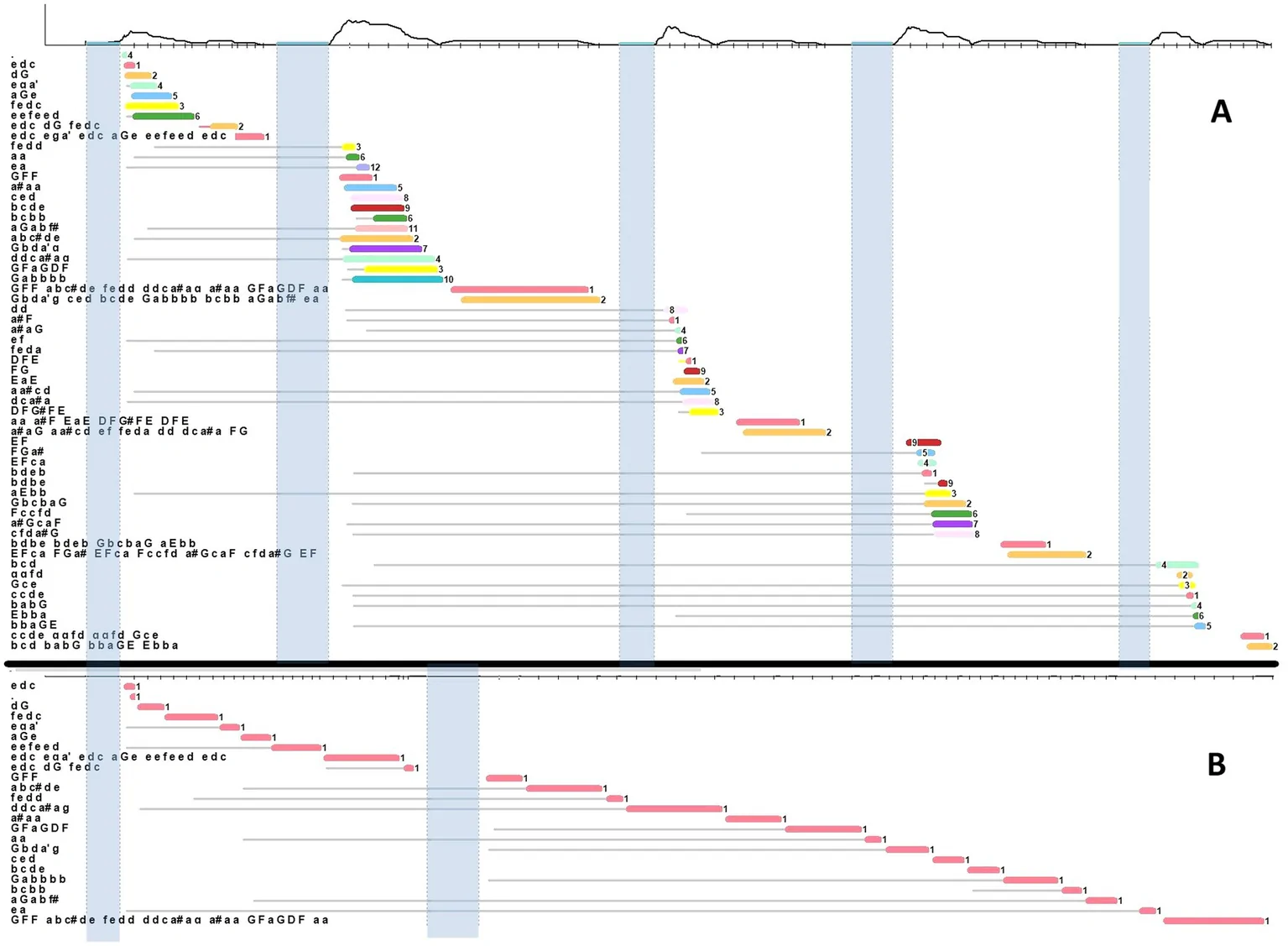
Parallel **(A)** or serial **(B)** treatment of *Hpc* replay. In both cases, *Hpc* here contains two active slices and can therefore encode two complex trajectories during online sessions (areas in light blue). **(A)** Offline, the concurrent formation of *Ctx* paths is based on the generation of a new carrier wave when *Ctx* sprouts a new branch, as long as a free wave is available. The curve at the top displays the number of waves used over processing steps, with each mark along the time-axis indicating when a replay cycle is launched by *Hpc*. **(B)** With a single wave, the same integration of new trajectories is serial, and its duration is significantly stretched: once the parallel process **A** is completed, 2/3 of the trajectories remain to be encoded through the **B** serial treatment.

The *Ctx*-*Hpc* interactions described above have been implemented in GP software. Computer trials reported below involve up to 50 elementary trajectories in the form of musical motives that are combined to create 10 full melodies. On completion of a *Ctx* memory path, the software labels its final unit by concatenating the more elementary components labels, which permits to check the accuracy of the learning algorithm. In terms of growth rate of a given population of paths, computer experiments notably show the benefit of building several *Ctx* path in parallel (Figure 6) as compared to a sequential computation (Figure 7). Whether or not physiological feature exist that would allow concurrent paths to develop simultaneously without interfering is to be explored.

## 3 From modelled functions to neurobiological structures

In this theory of memory formation, unused functional units become the meeting spot of branches presumably grown from the existing cortical hardware. Owing to a learning prerequisite, this basic script is restricted to offline time frames, a challenging feature considering the growth rate of neurites: depending on the nerve type, the extension of axons varies in the range of 20–75 μm/h, with a 100 μm/h velocity peak (Lallemend et al., 2012). Additional data must therefore be called upon to investigate whether structural/anatomical counterparts of the theoretical model would satisfy relevant temporal constraints.

### 3.1 Possible concurrent guidance of axonal sprouting

Every time a given sequence is produced by *Hpc*, its *Ctx* private path becomes fluently activated unless an unexpected stimulus occurs that initiates the sprouting of a new bud towards an available target unit. A later repetition of the same sequence reinstates the contextual flow up to the point where the relevant path is being built, one more step towards the target. If trajectories are quickly played *one after the other*, their total number determines the time interval between two subsequent repetitions. However, even when many trajectories are involved, the linkage of neurites between an existing unit and its free target is unlikely completed within such a single interval. Molecular insights into brain development and repair may shed light on this key issue.

Injuries to the nervous system, such as stroke, tend to bring about repair mechanisms (Carmichael et al., 2017). Brain rewiring (e.g.: in the visual cortex: Majewska and Sur, 2006) faces the issue of accidental gaps to be filled, also raised by the outgrowth of GP memory paths between distant units (e.g.: *A* and *B*). For regeneration purposes, glial cells acquire a bipolar morphology and together form a bridge that guides axonal extension from *A* to *B* (Rigby et al., 2020). Not only do glial cells show the right way through their orientation, but they also express *guidance cues* that are able to bind receptors present in the *growth cone* at the axon tip (Domínguez-Romero and Slater, 2021). However, few canonical molecules have been identified as *attractant/repellent* cues in animal species with a brain, which does not allow the full cortical connectivity to result from genetic programming. Despite the consequent difficulty for brain axons to reach pre-planned targets, scientific reviews show a trend to overlook ionic variations for coping with the lack of guidance cues (Stoeckli, 2018). Yet the versatile Ca^2+^ calcium ion may notably be regarded as a key player in channelling axonal extensions, supposed here to mostly occur during SWS. This possibility seems contradicted by the homogeneous downregulation of calcium across sleep, presumably linked with the downscaling (i.e.: re-normalization) of synapses (Tononi and Cirelli, 2014). But unexpectedly, in sleeping mice, local cortical circuits have recently unveiled a distinctive calcium upregulation during SWS bursts of oscillatory activity (*Sleep spindles*), occurring furthermore in layers 2–3 of special interest here, supporting the idea that “during sleep, and SWS in particular, an upregulation occurs in cell assemblies which were specifically involved in prior encoding of information, while network activity is globally downregulated” (Niethard et al., 2021). Delving further into knowledge relating to Ca^2+^ may clarify its involvement in axonal guidance.

Depending on whether Ca^2+^ is expressed at a low or high range inside the growth cone of an axon, the latter either expands forward or retracts (Henley and Poo, 2004). Among potential directions taken by the *A* axon, this property favours the one that satisfies the highest Ca^2+^ concentration induced by an extracellular source (Sutherland et al., 2014). Additionally, interstitial glial cells (i.e., *microglia*) regulate neurogenesis and brain development in neurons that are still devoid of synapses, therefore “assisting activity-dependent neuronal integration” (Cserép et al., 2022). Because *astrocytes* can convey intercellular Ca^2+^ waves named *glissandi* (Kuga et al., 2011), glial cells can be suspected to form a *gradient tunnel* between the *A* axonal growth cone and *B* dendrites, reminiscent of neural stem-cells migration (Kaneko et al., 2017). Accordingly, neural migration and axonal sprouting would share steering principles. To proceed towards experience-driven targets (Devreotes and Horwitz, 2015), either full NSCs or their only extensions may therefore be influenced by attractors and repellents, including Ca^2+^ (D’Arcangelo, 2014). Calcium signals are indeed evoked by neuronal activity within glial cells, generate a spreading intercellular Ca^2+^ wave, and initiate responses in adjacent neurons (Deitmer et al., 1998). An experience-dependent guiding can thus operate: Given its particular dual sensitivity to calcium levels, the *A* cone likely expands on learning demand towards the direction that ensures the highest calcium level and retracts otherwise when its intracellular Ca^2+^ does not receive significant external input.

Besides, the parallel growth of different axons can even be contemplated when realizing that Ca^2+^ waves may support the temporal coding already applied to the concurrent storage of *Hpc* trajectories. Different characteristic phases/frequencies could distinguish concurrent waves of calcium spikes and therefore avoid mutual interference between simultaneous extensions of axons: In the same way that a generic path can withstand several records without cross-interference, axonal pathfinding would then be committed to distinct waves of calcium spikes in order to avoid irrelevant cross-connections between *Ctx* concurrent paths (Figure 6). In support of this setting, a major second messenger known as cyclic AMP participates in the local production of brief Ca^2+^ transients in cone extensions, allowing “different downstream effectors to be sensitive to different frequencies” (Nicol et al., 2011) while steering the axon (Forbes et al., 2012). In addition, the local generation of Ca^2+^ in the form of “non-bursting regular spiking firing” (Magó et al., 2021) has recently been found in layers 2/3 of the human cortex (Gidon et al., 2020). Synchronous waves could thus be triggered and sustained through repetitive replay until neurites locked to the same frequency/phase form synapses. Owing to the limited growth rate of axons, large spikes embedded into calcium waves should induce sustained activation long enough for the *A-B* distance to be covered, a process reminiscent of LTP, which is usually thought to only govern the setting of synaptic weights. Interestingly, high-amplitude Ca^2+^ triggers attractive steering of the cone and induces LTP, whereas low-amplitude Ca^2+^ is involved in both repulsive effects and LTD (Henley and Poo, 2004), implying that similar regulation could affect both axon outgrowth and subsequent synaptic setting. Not only restricted to priming synaptic consolidation in neural assemblies, the present theory therefore suggests that Ca^2+^ may primarily steer axonal sprouting.

In the “physiological learning” scenario suggested here, pathfinding would be performed during the slow-wave activity (SWA) of sleep cycles, often viewed as the hallmark of N3 (SWS) stage. SWA was however recently precisely quantified including during N2 wherein Sleep spindle contribute to memory consolidation (Xu et al., 2025). Although affected by ageing (Rosinvil et al., 2021), SWA total duration is rather stable over a lifespan, approximating 45–50% of the total sleep time (Guo et al., 2022). Given that the average neurite growth rate can be estimated to equal 50 μm/h, SWA summed over 5 sleep cycles would therefore enable at most 7.5/2*45 = 170 μm of axon elongation, albeit for as many parallel axons as distinct Ca^2+^ “carrier waves” presumed to spread in parallel. A significant metabolic enhancement can be expected then. Sleep studies have indeed shown that the synthesis and decomposition of brain lipids are both greater during sleep than during wakefulness (Aalling et al., 2018). Also in keeping with the offline outgrowth of layer-2 neurons, both SWS stage and Growth Hormone (GH) secretion are promoted by the GH-RH hormone (Obal and Krueger, 2004), and a significant portion of the total daily GH secretion occurs during SWS (Van Cauter and Copinschi, 2000). In particular, GH peaks are known to rise minutes after the onset of SWS, and affect nervous tissues that possess GH receptors, thus inducing axonal elongation (Baudet et al., 2009). Although resident stem cells responsive to GH have been found in the adult brains of mice (Blackmore et al., 2012), large-brained mammals have not yet been subjected to similar investigations. Overall, immature layer-2 cells may be sensitive to the selective production of GH during SWS, which is conducive to their proper extension, on conditions already outlined, namely both segregation of cortical areas against proactivity and reduced internal/external perception to prevent interference. The Brain-derived neurotrophic factor (BDNF) may also be involved, since its concentration is inversely correlated with mnemonic symptoms in several psychiatric conditions (Miranda et al., 2019). Additionally, *neuritin* was recently identified as mediating the outgrowth of neuromodulating neurons (Shimada et al., 2024) that should accompany adult neurogenesis in the hippocampus.

Behavioural data on sleep also underpins the role of Ca^2+^ in memory. When the coincident occurrence of Ca^2+^ is disrupted in mice, the latter unexpectedly shows elevated anxiety and memory impairment (Gangarossa et al., 2014). This observation indicates that Ca^2+^ is instrumental in memory-related physiological events associated with sleep spindles. Notably, massive Ca^2+^ entry by these bursts of neural oscillatory activity has been proposed to “*open the door to subsequent long-term changes in cortical networks* (…) *during a state in which sensory processing should not occur*” (Sejnowski and Destexhe, 2000).

### 3.2 Matching the model architecture with thalamocortical structures

“Economy of wiring” has been a leading concept in the search for very local groups of cells in the cortex (Hubel and Wiesel, 1962). The human cerebral cortex is, on average, 2.5 mm thick, with regional variations ranging from 1 to 4.5 mm (Fischl and Dale, 2000). Perpendicular to the layers of the cortex, the near-cylindrical *cortical column* can be regarded as a toolbox, the essential attributes of which are geared to its cortical area. This view has infused the more recent notion of a “*canonical circuit*” (Douglas and Martin, 1991), referred to as *canonical cortical circuit* (CCC) in the following.

Instances of CCCs can be distinguished by their cell types, layer thickness, and receptive field properties, whereas the core circuitry notably controls the stimulus income and the excitation/inhibition balance (Da Costa and Martin, 2010). Of special interest here is the integration by CCCs layer-4 of two inputs, one issued from layer 2/3 of an upstream CCC, and the other one from *thalamic* stimuli. A parallel can tentatively be drawn with the dual input of a GP unit, whereby the latter can be activated on the one hand by contextual information conveyed by upstream units of the same storey and, on the other hand, by stimuli from the more peripheral storey. Mutual inhibition, as implemented between GP units primarily for resetting lapsed anticipations, is performed by inhibitory “layer 2/3” interneurons across CCCs (Helmstaedter et al., 2009). The neuroscientist who discovered cortical columns left half-open the question of their remote relationships by stating that “Columns in cytoarchitectural areas located at some distance from one another, but with some common properties, may be linked by long-range, intracortical connections.” (Mountcastle, 1997). A more specific organization principle has emerged from psycholinguistic considerations, including *priming* effects in the treatment of sequences, suggesting a way for columns to anticipate thalamic stimuli. A primed/called column would seek to integrate expected stimuli, hence the notion of “call-tree” at the core of a cortical model (Burnod, 1988; Alexandre et al., 1991). A call-tree resembles a GP module that has completed the differentiation of several paths. With respect to biological inspiration, the call-tree neurobiological model originates from data relating to the cortex organization, whereas the GP functional model was initially only inspired by the summation of *post-synaptic potentials*. Issued independently in the 80s’, developed in different domain areas and based on distinct data, similarities of the resulting architectures are nevertheless indubitable. Now focusing on the learning script specific to GP, whether cortical and thalamic trees can offline be driven to meet at the entrance of CCCs is to be considered. In this regard, what fosters further anatomical review is the possible integration into operational circuits of free CCCs targeted by extensions of layer-2 of an upstream CCC.

### 3.3 Neural growth with constraints

**Figure 8:**
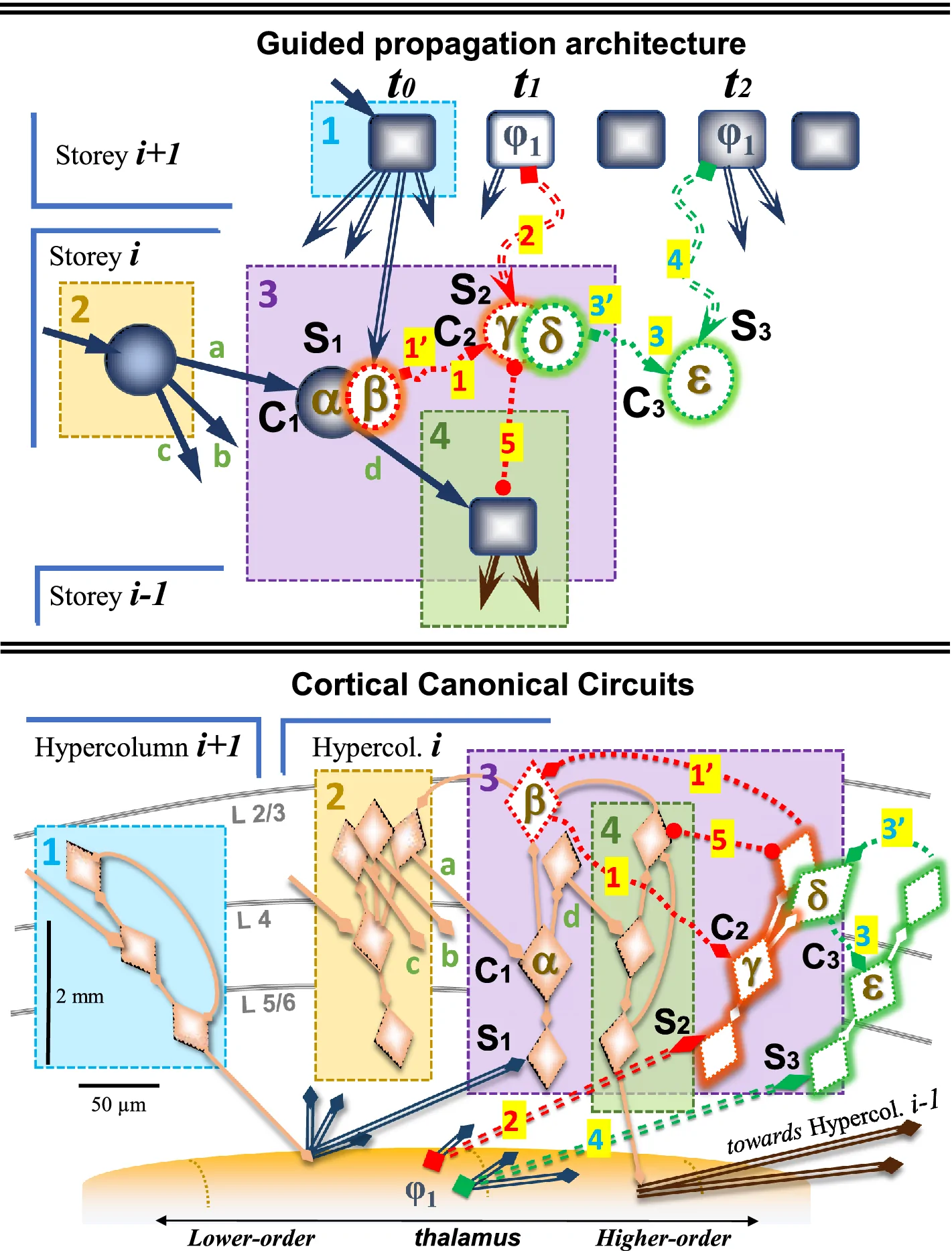
Tentative mapping of the GP topology (upper window) onto thalamocortical structures (bottom window). Colored numbered boxes show the match between the two structures’ elements: GP units resemble CCC radial structures composed of pre-wired internal connections between neurons (diamond-shapes). Both representations emphasize the contextual (C_1_, C_2,_ …) and stimulus (S_1_, S_2_, …) inputs of functional units. Whereas C_1_ feeds unit *α* of layer 4 (L4)_,_ S_1_ feeds layer 5/6 of the same CCC. Here, the unprecedented coincidence of C_2_ and S_2_ induces the recruitment of a layer 2/3 dormant unit (*β*, in dotted red) that will branch to layer 4 of a free column (shadowed in red within box 3). The ongoing growth of a memory path, stamped with a new carrier-wave (i.e.: φ_1_), goes on by integrating another free (dormant) unit (*δ*), as well as another free CCC (shadowed in green). Novel links may be either activating (1, 2, 3, with an arrow head) or inhibiting (lateral inhibition 5, with round heads). Double straight lines at the bottom represent the wiring stemming from the *thalamus* (fed by sensors/effectors of several hypercolumns, such as displayed in blue box 1 and green box 4). Crosstalk between *hypercolumns* akin to GP modules of different storeys/channels is mediated by distinct thalamic territories (separated by brown boundaries at the bottom). Of note, both upstream facilitation and downstream activity are represented by a single link in the GP display, which corresponds to a couple of links with opposite orientations between CCCs (onwards activation 1, 2…, and backwards facilitation 1′, 3′…).

In the brain, the hippo-cortical interplay is likely mediated by a hub-like structure, be it either thalamic (Hwang et al., 2017; Yang et al., 2015) or cortical, such as the *subiculum retro splenial pathway* (Nitzan et al., 2020). Neurons of layers 5/6 are reciprocally connected to the *thalamus*, whose primary role in transmitting sensory signals likely extends to the relay of information across cortical regions (Sherman, 2017; Nakajima and Halassa, 2017) with the selective facilitation/gating properties of a *connector hub* (Shine et al., 2023; Jitsuishi and Yamaguchi, 2025). From a functional point of view, the tips of GP paths are similarly meant to interface different modules, possibly belonging to adjacent storeys that represent nested levels of hierarchized patterns. Additionally, a GP module is dedicated to treating a certain cluster of stimuli, which echoes the definition of a *hypercolumn*. Given that units at the interface between modules undergo the same cyclic modulation for learning purposes (i.e.: threshold drop for detecting events that have not been reset by the ongoing inner propagation), their grouping within the same structure reminiscent of the thalamus appears beneficial. Similarly, a memory path could project its output towards other hypercolumns via a thalamus-like region. The GP multi-storey multi-block building then becomes flattened while keeping its original topology, forming a mosaic of hypercolumns connected by thalamic nuclei (Figure 8).

With respect to the C contextual input to functional units, forming cross-connection between CCCs should comply with the growth rate of axons. The thickness/diameter of a CCC is at most on the order of 25–30 μm (Da Costa and Martin, 2010), which is also the distance separating two neurons located along the central axis of their respective adjacent CCCs.

This global calculation implies that the development of downstream/cross connections from a given CCC would first enlist adjacent CCCs. In the adult cortex, layer-2 NSCs could therefore only plug in the remaining free CCCs, behind the full set of those already reached by layer-3 neurons at an early stage of development. Since both short- and long- distance connections likely depend on the originating layer, either the 3rd or 2nd layer (Vasković, 2023), the consequent learning-dependent disparity could explain the non-systematic distribution of responsive CCCs that bewildered their supportive scientists. By implication, the older the adult cortex, the more distant the CCCs that are not already functional, the fewer new links possibly built up overnight, and the more time-consuming the full storage of long-term memories. Unless adult neurogenesis compensates for the depletion of free-CCCs.

Approximately four months would be needed for thalamocortical axons to develop in the human brain (Željka et al., 2017), which entails limited neurite extension from the adult thalamus for adaptive purposes. In Figure 8, the S input to L 5/6 would therefore selectively consolidate the involved connections among many already present, which recalls the classical synaptic learning.

### 3.4 Towards adaptive processes encompassing sleep–wake constraints

During sleep, axonal sprouting from layer-2 stem cells is supposed here to govern the cross-linkage of CCCs. Additionally, computer simulations show that the outgrowth of GP paths can be steered in parallel (Figure 6). To this end, a novel “Ca^2+^ wave” is bound to the sprouting of a branch that encodes a new trajectory. By referring to a function of the GP algorithm, thalamocortical signalling could ensure the detection of unexpected thalamic activities owing to the absence of cortical ‘reset’ feedback whenever a (thalamic) stimulus fails to coincide with a (cortical) contextual signals. In this case, unexpected stimuli are not reset, and can therefore be identified as the only ones that respond to a depolarizing signal triggered regularly. Provided the availability of a carrier wave, an extra branch (C) extends towards layer-4 of a free CCC from layer-2 of the last activated CCC. Meanwhile, the layer-4 CCC is concurrently activated by the thalamic output tuned to the same wave. This scenario is compatible with an “instructive” role of the thalamus, even “in late network development that persists into adulthood as substrate for learning and memory” (Sur and Leamey, 2001). Blind axonal extension along a preferred direction, repelled by concurrent travelling waves attracted by free CCCs (e.g., chemical attractants or Ca^2+^ waves concurrently brought by thalamic afferences), may channel this search fed by spontaneous activity. Nevertheless, whether the development of cortical maps is driven by inner neural activity or the thalamus remains open to conjecture (López-Bendito, 2018). If thalamocortical connections are pre-wired while inter-column links develop gradually over sleep cycles, one is inclined to believe in the precedence of thalamic signals over cortical ones, although both contributions are required.

Computer trials performed in this study are paced by two encoding cycles, irrespective of the actual circadian timing of sleep:

1. Online labile encoding of unexpected patterns, as long as free *Hpc* slices are available, as well as phases within each slice. The *Ctx* channel is concurrently activated for notably relieving *Hpc* from the storage of already known trajectories.

Switch to:

2. Offline when *Hpc* learning resources are exhausted.

- *Hpc* replay of patterns bound to previously recorded melodies.
- Updating of *Ctx* memories with *Hpc* unknown trajectories.
- Replay of each just-completed *Ctx* path for creating a cross-connection with the *Hpc* path which is thus activated slightly later.

3. Switch to online encoding (1/) once every *Hpc* memory is represented into *Ctx*. *Hpc* slices and phases must be recycled unless newborn slices can be brought into play.

Currently, switching from one process to the other occurs as soon as encoding is finalized within a channel, either *Hpc* or *Ctx*, regardless of the actual duration of sleep cycles. As stated above, the formation of new *Hpc* memory paths is associated with a growing scarcity of units at disposal, together with the poor recycling of distinct carrier waves. In the computer experiments reported in Figure 7, the completion of *Ctx* paths is indeed greatly delayed when a single wave is involved.

### 3.5 Quantitative aspects

Within the framework of this theory, the building of cortical memory paths relies on several parameters with known ranges, including the axonal extension rate, the distance between CCCs to be linked, and duration of the SWA neural growth period. Otherwise, involved parameters may either be speculated or possibly informed by current data. A notable exception is nonetheless the timeline necessary for a quiescent cell to be awakened beforehand (Blasco-Chamarro and Fariñas, 2023).

Among known biological variables, the growth rate (*GrowthRate*) of axons spans from 20 to 75 μm/h and may peak to 100 μm/h. Since the distance separating the centre of two cortical columns can be averaged to *d_CC* = 30 μm, the travelling time of an axonal tip from a given CCC to a close neighbour goes from 24 mn (30 μm/75 μm/h = 0.4 h) to 90 mn (30 μm/20 μm/h = 1.5 h), and may be as short as 18 mn (30 μm*/*100 μm/h = 0.3 h), which is compatible with the SWA duration (around 45 mn per sleep cycle) (Guo et al., 2022) enabling a cross-connection to be achieved with 40 μm average *GrowthRate*.

On the hippocampus side, the model introduces *Nb_HpcSli* generic paths (i.e.: slices) and *Nb_HpcPha* characteristic phasic signals for each slice, which gives the *Hpc memory capacity*: *Nb_HpcSli* × *Nb_HpcPha*. On the cortical side, the model puts forward *Nb_CtxWav* carrier waves for simultaneously building private paths that link CCCs.

If *Nb_HpcTraj* trajectories are to be learnt, the set of *Nb_CtxWav* carriers will be called *Nb_HpcTraj / Nb_CtxWaves* times. The time spent growing a trajectory segment between two CCCs is *Nb_crossCC* × *d_CC / GrowthRate*, where *Nb_crossCC* is the number of already used columns to be crossed before reaching a free one.

Let *T_growth* be the time for growing *Nb_Traj* trajectories, each made of *NbPathUnits*, with the following variation intervals: [1, 10] for *Nb_CtxWav,* [1, 5] for *Nb_crossCC,* [25, 100] for the *GrowthRate,* [3, 7] for *Nb_PathUnits.*

*T_growth* = (*Nb_Traj/ Nb_CtxWav*) × (*Nb_crossCC* × *d_CC / GrowthRate*) × *Nb_PathUnits.*

In order to build at an average rate of 40 μm a long trajectory of 5 units without parallel processing (*Nb_CtxWav* = 1) between close CCCs, *T_growth* = 18mn × 5 = 90 mn = 2 SWA cycles, which means that 6 sleep cycles are required in these conditions. One way to increase the encoding capacity of *Ctx* is to increase *Nb_CtxWav*, even though the theoretical dividing of the processing time by *Nb_CtxWav* cannot be obtained with a sequential algorithm, as shown in Figure 7 that only exhibits a 2.5 reduction of *T_growth*. With this time saving, 7.5 new trajectories could come out of a 9-h sleep.

*Nb_crossCC* is a limiting factor of *T_growth*, emerging in the mature memory system that has already depleted the pool of free and nearby CCCs accessible from any CCC.

Among the parameters introduced above, both SWA integrity and axonal growth bear the greatest responsibility for the possible fall of cortical memories behind those replayed by the hippocampus, as illustrated by a study of depression (Béroule, in press). As *Nb_crossCC* increases with ageing, the formation of new memories would not only be compromised by a decline in SWA (El Kanbi et al., 2025), but also by the longer ride of neurites between busy and free columns.

## 4 Autism spectrum disorder revisited

Many features of unknown origin and variable strength characterize the mental disorder referred to as autism (ASD), starting with the riddle of an exponential increase of cases since the 1970s. The augmented recognition of ASD cases, implying more reporting and application of diagnosis (Castro et al., 2026), is believed to contribute to a social burden assimilated with caution to an epidemic (Wazana et al., 2007).

### 4.1 A slowly exponential and worldwide emergence

Autism is a global threat. While a prevailing cause of heterogeneity in the estimates points to country income and development, with higher prevalence in the US (1.12%) and Sweden (0.9%) (Talantseva et al., 2023), this explanation is challenged by lower estimates calculated for instance in France (0.35%) over the same 1994–2019 period.

A 787% gradual increase in incidence of autism diagnoses over one generation in UK has recently been witnessed by an *index number* updated every year from the 100% baseline index in 1998 (Russell et al., 2022). In its latest survey also conducted in 2022, the *Autism and Developmental Disabilities Monitoring Network* reported another prevalence indicator that recently reached 3% of US children, a value 4.8 times higher than the 0.6% estimation performed at the beginning of the century, while acknowledging an increasing rate of ASD identification (Shaw et al., 2025). Variations of ASD prevalence appear to be as unexpected as in actual epidemics, but rather than showing a sudden spread in the number of cases from a certain location, it grows slowly and worldwide. As compared to the rapid propagation of a single infectious agent, could this gradual and widespread emergence be imputable to the summed co-occurrence of environmental factors across human generations?

Most severe children disabilities are associated with up to 150% oversized brain (Davis et al., 2008; Keown et al., 2013), followed by an accelerated rate of volume decline in adult patients (Courchesne et al., 2011a, 2011b). Lower connectivity between distant regions such as the parietal (primary perception) and frontal lobes (executive functions) can be observed together with an increased local connectivity, albeit not in every report (reviewed in: Mohammad-Rezazadeh et al., 2016). Additionally, a definite rise of symptoms appears lately after birth, around 2 years of age (i.e.: *regression*), and boys are at least 4 times more affected than girls (*sex ratio* = 4:1). Among siblings, 20% of newborns with an older autistic sibling developed ASD, consistently with the prevailing wisdom on the part played by genetic factors. Last but not least in the context of the present study, poor sleep is often reported, though more often as an aggravating factor than as the cyclic impaired activity that could underlie brain development deficits. Recent analyses indeed support the causal role of insomnia in ASD (Wintler et al., 2020; Albertini et al., 2025). Up to 86% children diagnosed with ASD fall asleep in more than 30 min (*sleep onset latency*), and suffer from frequent night awakenings along shortened sleep relative to typical development. Although still barely prescribed, visualization of the sleep architecture by *Polysomnography* could therefore provide hints on the severity of ASD with an adapted protocol (Ezedinma et al., 2025), as for neurodegenerative diseases (Antonioni et al., 2025). Whilst disclosing early epileptic signs that justify the prescription of an anticonvulsant, the macro- and micro- structure of sleep revealed by polysomnography show significant *wakefulness after sleep onset* associated with a decrease of spindle density and duration as compared to controls (Petruzzelli et al., 2021).

Along the enzymatic avenue that may explain the above findings, a deficit of melatonin-related enzymatic activity has been shown to induce low concentration of melatonin, hence impairing circadian rhythms in ASD (Gillberg et al., 2008). Similarly, an unbalanced degradation of monoamines could delay sleep onset, extend micro-awakenings and delay the onset of paradoxical sleep, hence impeding the lengthy formation of cortical memories across fragmented SWA (Béroule, 2018). The physiological dysregulation at issue can be characterized as *epigenetic*, for it responds to the pressure of ongoing events by changing the expression of genes. But so far, research has mostly tried to identify irreversible mutations at the basis of a disorder presumably rooted in the family lineage. Because each mutation thus detected only accounts for about 2 % of ASD cases (Betancur, 2011; Miyauchi and Voineagu, 2013), cumulative effects of multiple mutations are likely required for the disorder to develop. Although the alternative of reversible epigenetic impact can be thought to follow the same collective effect (Herrera et al., 2024), strikingly the shifted expression of a single gene could reveal disrupting enough at the foreground of genetic variants, while being possibly induced by several risk factors.

### 4.2 Autistic features within the same theoretical framework

At least one particular gene stands at the bottom of a funnel fed by a large set of promoters. Whether chronic exposure to *fatty acids* (SCFA) such as *valproic acid* (VPA; Zarate-Lopez et al., 2024), heavy metals (cadmium, lead, mercury, as notably found in cigarettes; Abdelouahab et al., 2010), oestrogens, alcohol, or systemic diseases such as depression, alterations of the gut microbiome and inflammation, several persistent/repetitive events share the property of upregulating the same brain enzyme, namely *monoamine oxidase type A* (MAOA) carried by the X-chromosome (Béroule, 2018, 2020). Rather recently has the likelihood of neurodevelopmental disorders (autism, Asperger) also been associated with pre-obesity in any of the parents (Surén et al., 2014). This finding is not surprising viewed through the lens of the current theory, when knowing about the significant upregulation of MAOA in overweight persons (Sturza et al., 2019). Maternal depression likewise increases the risk of having a child with ASD. If the brain development requires a balanced metabolism of its monoamines, it seems logical that an accidental overexpression of MAOA triggers its early epigenetic downregulation in affected neurons of the foetus. After birth however, free of maternal blood income, MAOA stays downregulated in this subset of monoaminergic neurons, hence an insufficient clearance of their downstream synapses. Regarding circadian cycles, this situation is particularly impacting during sleep phases when the synthesis of both serotonin (5-HT) and noradrenaline (NA) is interrupted, which should normally cause their removal from the synaptic cleft under enzymatic action.

More than their common MAOA downregulation, discrepancy between monoamines concentrations can result from the concurrent action of other brain enzymes. Indeed, while 5-HT is not affected by the COMT enzyme, the latter alters both dopamine (DA) and NA to a variable degree that depends on a *genetic polymorphism* whereby the High-COMT (GG) phenotype is more effective. As another potential cause of monoaminergic imbalance, only DA is decreased by the MAOB whose blood concentration increases until about 2 years of age (Tong et al., 2013), with a polymorphism-dependent strength higher in its GG phenotype as well (Kakinuma et al., 2020).

This explanation is substantiated by the existence of factors that affect the expression of MAOA. Surprising as this may seem, most ASD symptoms can indeed be derived from a specific combination of MAOA downregulation and genetic variants of COMT and MAOB, taken together with the non-fulfilment of the GP learning constraint: Instead of gradually and equally decreasing with NA in order to support axonogenesis at the core of new skills development, 5-HT would undergo slower decay in the synapse over every sleep cycle, including its final PS when both monoamines should have fully been cleared.

#### 4.2.1 MAOB maturation and behavioural regression

Defined as a loss of verbalization when only a limited vocabulary is used by the toddler, ASD regression is associated with impairment of social communication at around 20 months of age (Al Backer, 2015). Individuals experiencing this early behavioural regression are more likely to have cognitive deficiencies compared to children without regression, as well as higher levels of autistic symptoms. Whereas the physiological origins of regression is still unknown, concomitant phenomena have recently been emphasized, such as a faster motor development (Manelis et al., 2020) and following trend to epilepsy and aggression (Gaitanis et al., 2023).

Over the same post-natal period that regression occurs, MAOB reaches its mature level for metabolizing DA. The shape of the GP “restricted propagation” area (Figure 1b) shows that an elevated *5ht/Na* ratio does not cross the border of this baseline area unless the excitability parameter is increased (i.e.: *Da*). In other words, a too-strong *5ht* may be ineffective as long as *Da* is kept high. Accordingly, regression could reflect excess serotonin revealed by a decreased level of dopamine especially when the “high MAOB” variant comes into play. Non-regressive subjects would undergo less severe symptoms later on, owing to variations of DA levels that cause irrelevant shift towards the “context-driven propagation” area (Figure 1b).

#### 4.2.2 Structural aberrations and behavioural deficits

When run with high *5-ht*/*Na* rather than the usual balanced ratio, computer simulations of offline memory integration tend to generate impaired memory traces that can then be compared with ASD behavioural deficits. Several outcomes were obtained by performing 50 online/offline alternations each time with a different *5-ht* noise. Apart from noise free-periods during which faulty memory paths stop growing, these are the cross modules that notably integrate emotional information and the deeper storey that are subjected to (up to 4 times) more aberrant connections than the peripheral storey. The number of correct paths undergoes acute drops as noise increases, starting by cross-circuits, followed by sensorimotor circuits that eventually incorporate 1.5 more units and 1.4 more inter-unit links. Besides, the same elementary trajectory can be integrated several consecutive times along the same memory path (see video: Béroule, 2016), thus producing irrelevant repetitions of this trajectory, mirroring the activation of short-range loops observed in *stereotypy* (repetitious and purposeless action).

The ASD “bigger brain” is therefore represented in this computational model, plus the emergence of many local and irrelevant connections that contrast with fewer long-distance connections as compared with noise-free simulations. Whereas a boosted local connectivity may elicit the amplification of perceptions, missing long-distance connections likely reflect a lack of control of active sensors (e.g.: eyes, basilar membrane of the auditory system), hence the impaired selection of useful stimuli. Additionally, distant connections include those that convey modulating signals for conducting decision-making through emotional control and acquired conditioning (Béroule and Gisquet-Verrier, 2016), acquired over PS according to the present model.

### 4.3 Preliminary trials and trans-generational epidemiology

Axonogenesis is argued here to support the formation of memories at a circadian pace, monitored by intricate patterns of neural signalling spreading across hippo-thalamocortical structures. The aim is to ensure neural growth in a context of low and balanced monoamines that avoids neuromodulation from cortical regions to burst into memory paths under construction. Although both 5-HT and NA subside over every sleep cycle, therefore implementing the sought balanced context, their degradation is handled by at least two enzymes (MAOA, COMT) whose distinct expressions can entail irrelevant discrepancy between synaptic concentrations of monoamines.

**Figure 9:**
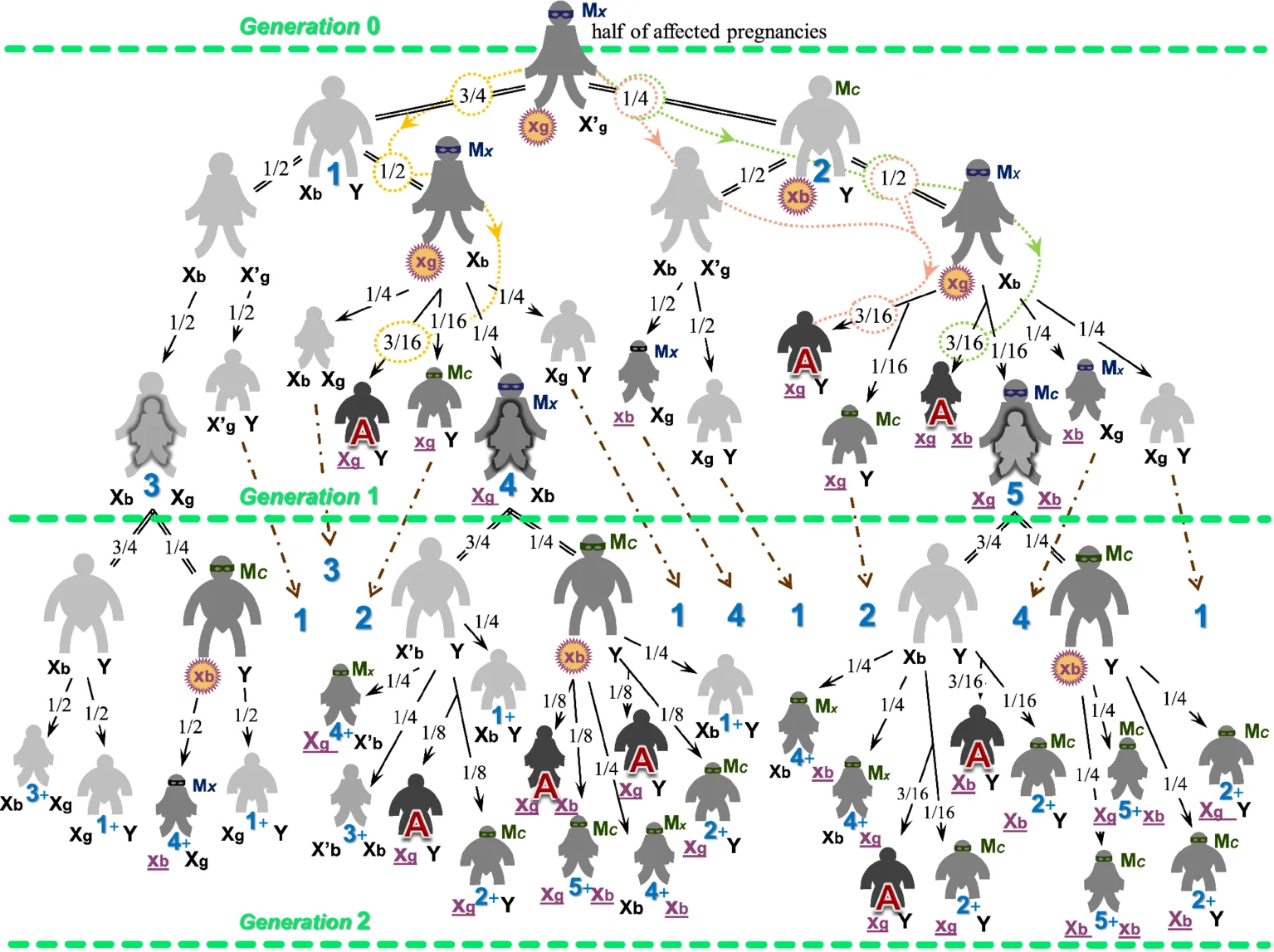
Family-tree wherein either overt or masked autism result from parents phenotypes affected through epigenetic downregulation of MAOA and genetic features. The full drawing is composed of 5 generative subtrees (labeled by blue digits), each defined by its position in the full tree, as well as by 5 distinct roots, namely icons of girl or boy, the darker the more affected. Severe non-autonomous ASD cases (labeled with a red “A”) are supposed not to have descendants. Otherwise, the likelihood of every generative branch is denoted by a unit fraction. The figure upper-half gives the offspring resulting from the first human generation within a given impacted community (excerpt from Béroule, 2018), and the bottom-half shows the spreading of the condition within a second generation. Although not drawn here, the third-generation distribution of phenotypes can be worked out from the 5 types of subtree roots at the bottom, coming up with the evolution of autism prevalence (see Supplementary materiall, section 2). Next to icons, M*x* and M*c* indicate masking effects of X-chromosome silencing and low-COMT, respectively. Xg, Xb represent X genes transmitted from a girl or a boy, underlined and redden when carrying disruptive traits, including for sleep (i.e.: low degradation of serotonin over every sleep cycle).

In response to the adventitious over-expression of MAOA in neurons during gestation, an epigenetic downregulation meets genetic variants of other brain enzymes as well as X-chromosome silencing in women, which notably accounts for the 4:1 sex ratio observed between boys and girls affected by overt/severe ASD (Béroule, 2018, 2020) as shown in Table 1. A former family-tree that combines relevant phenotypes is resumed and extended here to four human generations with the goal of deciphering the evolution of ASD prevalence (Figure 9).

**Table 1:** Likelihood of 7 female (X, X) and male (X, Y) phenotypes generated by 5 family subtrees, and their evolution over 4 human generations (right-most columns).

Phenotype		(X, X)	(X, Y)	(X, X)_MX_	(X, X)_MC_	(X, Y)_MC_	Generations				Subtree	3	1	4	5	2	First	Second	Third	Fourth
Unaffected	(X, X)	3/8	3/8	3/16			28	28	26	25
	(X, Y)	1/2	3/8	1/4		3/8	37,5	34	33	40
X silencing	(X, X)_MX_	1/8	1/8	1/4	3/8	3/8	18,7	14	13	14
Genetic masking (Low-COMT)	(X, X)_MC_			1/32	1/8	1/32	0,8	0,8	6	13
	(X, Y)_MC_		1/32	1/8	7/32	1/32	3,1	3,8	3,1	4
Overt autism	(X, X)_A_			1/32		3/32	2,3	0,9	0,8	**0,7**
	(X, Y)_A_		3/32	1/8	9/32	3/32	9,4	6,4	3,4	**3,6**
Male prevalence							4	7	4	**5**

The X symbol stands for a X chromosome affected by a MAOA downregulation. MX and MC suffixes represent the masking effect of a X-chromosome and the low-COMT variant, respectively, whereas the “A” suffix indicates overt/severe autism. For a given human generation, the ratio between (X, Y)_A_ and (X, X)_A_ gives the male prevalence.

Prior to that, new elements have come to complement this theory since it was formulated, including in hindsight, the alleviation of severe cases of autism in children having been prescribed a drug which promotes MAOA.

#### 4.3.1 Valproate prescription and re-education program

One case of severe autism with chronic insomnia has been followed for one year, with the aim of assessing the prediction that a supply of MAOA would improve sleep, and hence the capacity to efficiently form memories (Béroule, 2019). The 11-year old boy received a classical drug aimed at children with epileptic signs detected beforehand by polysomnography. The child psychiatrist agreed to treat his patient with *Valproate* (VPA), a MAOA promoter, rather than with other possible antiepileptic drugs. Sleep improvement has been immediate, and accompanied over months by the emergence of relevant skills observed along several dimensions of the autistic spectrum by relatives and physicians unaware of the secondary effect sought by the anticonvulsant drug. However, a decade after the end of this follow-up, the young adult behaviour does not actually meet expectations based on improvements that arose at that time. Because of the lack of speech therapists and specialized structures, no re-education program could be conducted to hopefully favour imprints of well-formed and accessible memories. Instead, existing stereotypies have likely been consolidated through their overnight inner replay. Additionally, although the VPA prescription has been continued, physicians who took over from the former paediatric neurologist replaced a complementary psychostimulant (*methylphenidate*) by sedative drugs with contradictory pharmacological effect that enhances the depressing trend of VPA, hence the onset of aggressive behaviour (Verdolini et al., 2017). Today, rare bursts of aggressively and few stereotypies are present in the young adult. Nevertheless, poor spontaneous language expression does not preclude obedience to parents, which implies understanding basic instructions.

By way of general observation, unsatisfactory progress may result from other factors than unsuitable medical and educational monitoring, considering in particular the significant changes undergone by the brain chemistry across adolescence (Carr and Ensom, 2003). Besides, the acquisition of language encompasses critical development stages, and late intervention in the pre-teen may not overcome a speechless decade.

Recently, an official clinical trial involving 100 children was conducted with the aim of assessing VPA for the treatment of autism. According to a global rating scale of ASD, global improvement of behaviour arose in 80% of the treated subject, as compared to 12% in the control group (Aliyev and Aliyev, 2018).

#### 4.3.2 Evolution of ASD prevalence

A trans-generational calculation (see: Supplementary material, section 2) can be derived from a former family-tree developed over two generations (Béroule, 2018).

**Figure 10:**
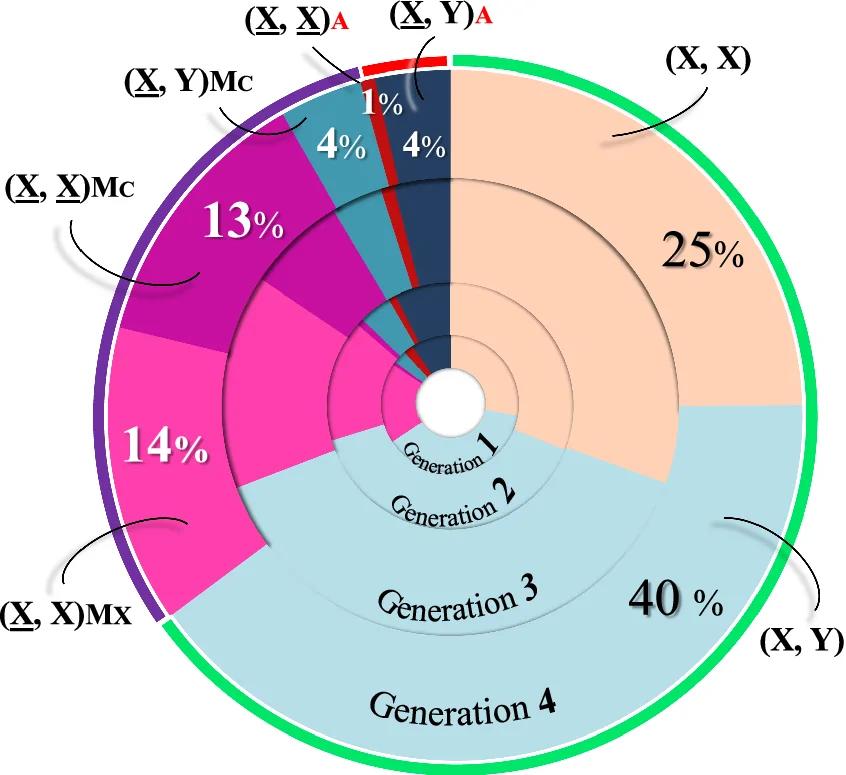
Distribution of genotypes relevant to autism across 4 generations of a population at risk. (X, X) and (X, Y): Non-affected girls and boys; (X, X): girls with one affected X-chromosome (underlined X); (X, X)_MC_ and (X, Y)_MC_: girls and boys protected by the low-COMT polymorphism; (X, X) and (X, Y): severe autism. Three external circular arcs show the proportion of individuals of the 4th generation whose X chromosome is not marked by an epigenetic regulation of excess MAOA (65% in green), those who are low-key carriers of the mark (30% in violet), and dependent persons (5% in red). The pattern remains stable across generation, save a significant rise in the COMT protection for girls.

In this calculation, the distribution of phenotypes remains remarkably stable over generations (Figure 10), save an increasing COMT protection for women and a significant decrease of severe autism (Table 1): in the subpart of the population initially affected, overt autism drifts from initially 2.3% to 0.7% in women and from 9.4 to 3.6% in men, while over the same four generations, the male prevalence would first grow from the classical 4 ratio to 7 before returning to 5. Consequently, an assessment of the ASD sex-ratio for a local population could be indicative of the period when this community started undergoing effective risk factors, and help identify the latter. The background spreading of low-key phenotypes likely enables the continuing emergence of disabling cases, and this, especially if the disruptive factors extend their territory. Whereas a tendency for more severe disabilities to slowly fade away is observed in this model, this prediction can be overridden by the possible cross-generation transmission of epigenetic marks once their driving factors have hopefully been terminated, and also through geographical extension of the affected population.

This examination of ASD spreading can be refined in several ways within the same framework. First, the family-tree should incorporate branches conveying MAOB variants, given their likely impact on the early regression process, and more generally, the depth of ASD deficits. Second, the binary clustering of either ‘overt’ or ‘low-key’ autism is to be confronted to symptoms that span from severe autism to hyperactivity via the Asperger syndrome. How could light ASD forms fall into the ‘silent autism’ clusters that contribute to the family-tree offspring? Third, by having recourse to geographical/nutritional/medical data, the expected outcome of epidemiological studies is to identify the summed toxins involved in the ASD origin, able to cross certain thresholds above which epigenetic reactions likely occur with sensitivity that may differ in different communities, consistently with the race-dependent ASD prevalence (Shaw et al., 2025). Fourth, it will remain to estimate how many generations would continue being impacted by the condition once its causing toxins have been identified and hopefully stopped.

By admitting the formation of cortical memory traces over the course of sleep, one is also led to acknowledge the need for proper neuromodulation over sleep cycles. A straightforward way to avoid an epigenetic downregulation of monoamine enzyme initiated during gestation would be to reduce beforehand the incriminated MAOA overexpression to its baseline level. Given the current extent of risk factors, systematic testing of MAOA can be recommended as preventive medicine for women with a parental project. As a first step, these factors should be lessened in conditions prone to recovery – though after months—such as infection, overweight and depression, as well as environmental toxins/drugs, including the *Valproate* anti-seizure medication and other SCFAs produced by the gut microbiome from food components. A possible maternal shift towards the production of SCFAs should be restricted prior and over gestation (Béroule, 2020). Overall, an individualized prescription of MAOA inhibitors could rectify deviations from the standard MAOA range, assumed here to be deleterious.

## 5 Synthesis and future prospects

It was more than a hundred years ago today, the pioneering work of Ramon y Cajal regarding the microstructure of the brain gave credence to his claim that “*once the* (embryonic) *development was ended, the founts of growth and regeneration of the axons and dendrites dried up irrevocably*”. Accordingly, only adaptive connections between neurons would support learning at the physiological level. This implication of Cajal’s words became a central doctrine of neurobiology and related computational models over the 20th century. Even years after the advent of the contradictory concept of adult neurogenesis, the birth of neural cells throughout life is still mostly considered useful for regenerating the brain in case of either acute injury or chronic disease. A more ambitious *neuroplasticity* is notably missing a vision of how, for the purpose of forming new memories, free neurons could integrate the adult brain without disturbing its ongoing functions. Towards a uniting solution, a specialized neural organ could stay focused on the recording of significant events while the rest of the brain keeps on running. Recognized in the adult nervous system as a recipient of strong neurogenesis, the hippocampus likely plays this role. But the problem of neural learning is thus shifted to the issue of how recorded data can become ‘knowledge’ through its integration into existing structures.

Sleep steadily interrupts the course of brain operations, moreover for a relatively long time; it is universally accepted as a period of necessary rest. Beyond this limited perspective, unconscious processes appear vital enough to justify lengthy and inescapable withdrawals from social activities at least on a regular circadian basis.

According to the theory introduced here, layer-2 quiescent cells are harnessed so that their axons grow in parallel towards free columns by means of distinct carrier-waves. Because these processes are sensitive to both proprioceptive/inceptive stimuli and inner modulating interactions, cyclic periods analogous to sleep prove indispensable, implying movement stoppage, withdrawal from the environment, as well as inner segregation of modules. More broadly, the speculative notion of offline experience-driven cortical growth, although untested yet, is bound to a series of embedded prerequisites that echo experimental findings: the sprouting of quiescent cells—conditionally steered upon the offline segregation of memories -, requires a recorder/player device for retaining online experience to be replayed offline, which necessitates rapid encoding as potentially supplied by the hippocampus signalling.

Apart from new insights regarding psychiatric conditions, the main outcome of this study is probably the enhancement of a topological mapping between the interacting modules of a memory model and cortical hyper-columns plus their thalamic connections. In this picture, hyper-columns are presumably crossed by spontaneous flows that tend to adaptively be locked to sequences of thalamic signals by means of the hippocampus replay function. The lifelong development of a hypercolumn tree-like structure is assumed to gradually incorporate free cortical columns at the growing points of new branches. Once a CCC is thus embedded for being committed to the integration of thalamic signals within a relative time-frame, its dormant cells are prone to extend their axon towards other unused CCCs to be involved in treating the next time-frame.

Sleep is shown to allow this cortical development scheme, not only because this is when online experience can be extensively replayed by the hippocampus, but also because it prevents the integration of replayed events from being spoiled by both external and inner signalling that occur in the waking brain, including motion-related ones and neuromodulation between brain areas.

Once added to the operational network, a CCC is ready to integrate the layer-2 output of an upstream column, while its own layers-5/6 can be solicited by an hyper-column via the thalamus. Accordingly, learning by differentiation would apply at the physiological level to hyper-columns, enabling their experience-driven development under adequate neuromodulation. The distance covered by an axon tip over the normal duration of SWA is shown here to fit with intervals separating cortical columns. With respect to connections with the distant thalamus, they may redundantly pre-exist and be selectively consolidated through the classical synaptic plasticity. As columns become more connected because of the accumulation of experiences, the growth process is led to target more distant columns, entailing extra time for completing the cortical storage.

Computer simulations helped to study “online instant recording” alternating with “offline repetitive replay” through well-synchronized interactions between *Hpc* and *Ctx*. Essential for the replay function, every GP memory path is versatile enough to either identify or generate embedded sequences (or trajectories) of discrete events. The production task is performed by a depolarizing wave that travels upstream a targeted path, which results in the activation of the path events, provided the priming of all the input/output beforehand. Given that this constraint concerns every type of sensor/effector, it applies to the most peripheral level of perceptive systems, including generators of eyes movements. Consequently, as a model prediction, paradoxical sleep would not only be associated with REM, but also with the activation of other perceptual systems at their periphery.

Several types of signalling between *Hpc* and *Ctx* could also be defined with the assistance of activity histograms plotted by the running software. On the *Ctx* side, these histograms reveal how local activations and modulations result in a global activation consistent with experimental findings. Although not yet examined in depth, the paradoxical-sleep replay of *Ctx* trajectories mostly aimed at connecting relevant *Hpc* path is shown to over-activate *Hpc* representations, which paves the way for addressing the generation of novel combinations of variables in existing episodic memories. This could either contribute to problem solving or facilitate the online management of unprecedented situations. In the present study, the replay of a just-completed *Ctx* path activates in parallel its *Hpc* generator, and the resulting simultaneous activity of both *Ctx* and *Hpc* paths endpoints elicits their cross-connection. This connection will afterwards prevent *Hpc* from unnecessarily spend resources for a trajectory already known by *Ctx*, and contribute to learning embedded and multi-representation patterns (Supplementary Material, Figure S2). Besides, *cortical replay* has been found to occur in all stages and cycles of sleep, ranging from thought-like to dream-like. PS would therefore harbour the same cortical replay than those triggered during other sleep stages, except that deeper, longer, embedded trajectories would then get activated.

The parallel growth of several *Ctx* paths was assumed to reduce the total time spent in emptying the “*Hpc* buffer.” But in order to avoid interference between concurrent paths, a new resource was exploited in the form of distinct calcium waves (Ca^2+^). Assessing whether the offline building of cortical memories is a valid assumption relies on technological developments that would permit to associate a new online experience with a discrete axonal growth in the concerned columns, which relies on their precise mapping.

Meanwhile, current functional limitations of the model should be reduced by delving into studies of additional brain sub-organs. Later extensions of the global memory model are firstly planned to incorporate *cerebellum*-like functions as an attempt to dynamically modulate sensorimotor patterns, likely through cortical inhibitory interneurons (Antonioni et al., 2024), as well as to handle the manipulation of variables (i.e.: distinct *Hpc* waves). Paradoxical sleep could then underpin the offline linkage of novel combinations of *slots* in known *frame structures* (steady generic paths) within parallel *Hpc* slices. Pattern recognition is intended to be addressed with varying *Hpc* waves by adapting online stimuli to their *Ctx* references, notably in terms of controllable spatial/frequency scales. Conversely, pattern production is expected to dynamically comply with motor requirements through the modulation of *Hpc* phasic patterns interplaying with *Ctx* backbone representations of trajectories. Together with the cerebellum, other structures could partake in the autonomous and sporadic encoding of new information. In reported computer tests, every online event activates both *Hpc* and *Ctx*, which enables the latter to send to the former a *matching score* for the ongoing trajectory (i.e.: likelihood of the best prediction). Owing to the elementary input data, all-or-none information was provided from a single *Ctx* channel. By integrating several brain regions, the *locus coerulus* could provide a more subtle “salience/surprise” income to the hippocampus than the current binary novelty indicator, whereby the ongoing activated representation could either be refined or fully encoded, thus raising new instances of sleep spindles (Groves et al., 2025).

Besides the replay of cortical paths aimed at forming connections with the hippocampus, paradoxical sleep is thought to create novel combinations of memories, most likely at the root of imagination that Henri Laborit (1970) expressed as being a “restructuration originale des expériences acquises”. When instances of time-coding apply to the manipulation of variables, the simulation of dream-like scenes will be expected to shed light on the role of imagination, formerly considered by Laborit as a privileged means of balancing unwise determinism and aggressive behaviour. Fifty years later, repressing hasty judgement while fostering novel conceptions has become a serious challenge for humanity to overcome the global threats it did create. More than ever, neurobiological knowledge is worth sharing and expanding, as it might notably contribute to “Give peace a chance”.

## Data Availability

The original contributions presented in the study are included in the article/Supplementary material, further inquiries can be directed to the corresponding author.

## Glossary

ASD: Autism spectrum disorder
BDNF: Brain-derived neurotrophic factor
Ca^2+^: calcium ion
CCC: Canonical Cortical Circuit
COMT: Catéchol-O-Methyl-Tranferase
***Ctx***: GP representation of a Cortical region
CX: Cortical region
DA: Dopamine
DCX+: doublecortin marker of immaturity
GG: High gene expression phenotype
GH: Growth hormone
GP: Guided propagation
HC: Hippocampus
***Hpc***: GP representation of a HC function
LTP: Long-Term Potentiation
MAOA: Monoamine oxidase type A
MAOB: Monoamine oxidase type B
NA: Noradrenalin
NSC: Neural stem cells
PS: Paradoxical sleep
REM: Rapid eyes movements
SCFA: short-chain fatty acid
SWA: Slow wave activity
SWS: Slow wave sleep
VPA: Valproic acid, Valproate
5-HT: Serotonin
