# Supplementary material for: The irreplaceable role of sleep in building long-term memories: offline integration of free cortical units and its possible implication in autism
Source: Front Hum Neurosci. 2026 Aug 14;20:1777687. doi: 10.3389/fnhum.2026.1777687 (PMC13521931; doi:10.3389/fnhum.2026.1777687)
Supplement: Supplementary file 1 [file Data_Sheet_1.docx]

***Supplementary material***

1. **GP system with several *Ctx* channels**

**Channels**

**Storeys**

**Slices**

**Cross-channel module**

**Figure S1**

GP network architecture. The *Hpc* channel (in green) contains here two storeys, while its slices develop dynamically in order to accommodate new trajectories. Sensors/ Effectors at the bottom of every *Ctx* channel (in yellow) are linked to *Hpc* (orange arrows) as well as cross circuits (black arrows) that allow modulation to be conveyed between modules.

**Figure S2**

Visual representation of the control of ***Ctx*** building by ***Hpc***. The extension of a ***Ctx*** module is conditioned by the availability of free cells, the stoppage of proactivity (“**Restricted propag**”), and the agreement of ***Hpc*** (“***Hpc* consent**”).

In the top frame, a compound pattern (**Storey *j-1***) cannot be built in ***Ctx*** before the completion of all its **Storey *j*** components that may not be stored in their serial order. The full activation of the corresponding ***Hpc*** path located in **Storey *j-1*** (to the left) allows its long-term version to be built in ***Ctx***.

In the bottom frame, paths that code for the same pattern in different channels must all be completed before they are connected by a cross-channel path. This occurs when the relevant ***Hpc*** detector has received signals from all its ***Ctx*** partners in different channels (red arrows). Online, the cross-channel circuits convey modulating signals.

**2. Detailed calculation of cross-generation of ASD prevalence**

Evolution of ASD prevalence across four consecutive generations of a phenotype family-tree that accounts for an epigenetic downregulation of the MAOA gene carried by a X-chromosome (written X in the following), together with the low-COMT gene polymorphism. MX and MC suffixes added to female (X, X) and male (X, Y) chromosomes code for the masking effects of X-silencing and low-COMT, respectively. The A suffix indicates overt/severe autism.

| **Phenotype** | **Subtree** | **(X, X)**  **3** | **(X, Y)**  **1** | **(X, X)_MX_**  **4** | **(X, X)_MC_**  **5** | **(X, Y)_MC_**  **2** |
| --- | --- | --- | --- | --- | --- | --- |
| unaffected | (X, X) | 3/8 | 3/8 | 3/16 |  |  |
|  | (X, Y) | 1/2 | 3/8 | 1/4 |  | 3/8 |
| X silencing | (X, X)_MX_ | 1/8 | 1/8 | 1/4 | 3/8 | 3/8 |
| Genetic masking  (COMT) | (X, X)_MC_ |  |  | 1/32 | 1/8 | 1/32 |
|  | (X, Y)_MC_ |  | 1/32 | 1/8 | 7/32 | 1/32 |
| overt  autism | (X, X)_A_ |  |  | 1/32 |  | 3/32 |
|  | (X, Y)_A_ |  | 3/32 | 1/8 | 9/32 | 3/32 |

**[** **] _n_** : Subtree **n**, with **n** є [1, 5]

**1^st^ generation**

3/4 x **[** **] _1_**

1/4 x **[** **] _2_**

**=>**

3/4 x **[** 3/8 (X, X), 3/8 (X, Y), 1/8 (X, X)_MX_, 0 (X, X)_MC_, 1/32 (X, Y)_MC_, 0 (X, X)**_A_**, 3/32 (X, Y)**_A_** **] _1_**

1/4 x **[** 0 (X, X), 3/8 (X, Y), 3/8 (X, X)_MX_, 1/32 (X, X)_MC_, 1/32 (X, Y)_MC_, 3/32 (X, X)**_A_**, 3/32 (X, Y)**_A_** **] _2_**

**=>** Likelihood of phenotypes:

9/32 (X, X), 12/32 (X, Y), 6/32 (X, X)_MX_, 1/128 (X, X)_MC_, 1/32 (X, Y)_MC_, 3/128 (X, X)**_A_**, 12/128 (X, Y)**_A_**

**=>**

**28%** (X, X), **37,5%** (X, Y), **18,7%** (X, X)_MX_, **0,8%** (X, X)_MC_, **3,1%** (X, Y)_MC_, **2,3%** (X, X)**_A_**, **9,4%** (X, Y)A

Sum of likelihoods: (4*(9+12+6+1) + 16)/128 = 100%

Sex ratio: likelihood(X, Y)**_A_** / likelihood(X, X)**_A_** =12/3 = **4**

No offspring: likelihood(X, Y)**_A_** + likelihood(X, X)**_A_** = 11,7 ≈ **12%**

**2^nd^ generation**

(3/16 + 3/32) x **[ ] _3_**

(3/16 + 3/32 + 1/16 + 1/32) x **[ ] _1_**

(3/32+ 1/16+ 1/32) x **[ ] _4_**

(1/128) x **[ ] _5_**

(3/128 + 1/128) x **[ ] _2_**

**=>**

9/32 x **[** 3/8 (X, X), 1/2 (X, Y), 1/8 (X, X)_MX_ **] _3_**

12/32 x **[** 3/8 (X, X), 3/8 (X, Y), 1/8 (X, X)_MX_, 1/32 (X, Y)_MC_, 3/32 (X, Y)**_A_** **] _1_**

6/32 x **[** 3/16(X, X), 1/4(X, Y), 1/4(X, X)_MX_, 1/32(X, X)_MC_, 1/8(X, Y)_MC_, 1/32(X, X)**_A_**, 1/8(X, Y)**_A_** **] _4_**

1/128 x **[** 6/16 (X, X)_MX_, 1/8 (X, X) _MC_ + 7/32 (X, Y) _MC_ + 9/32 (X, Y)**_A_** **] _5_**

1/32 x **[** 3/8 (X, Y), 3/8 (X, X)_MX_, 1/32 (X, X)_MC_, 1/32 (X, Y)_MC_, 3/32 (X, X)**_A_**, 3/32 (X, Y)**_A_** **] _2_**

**=>** Likelihood of phenotypes:

(X, X) : 9/32 * 3/8 + 12/32 * 3/8 + 6/32 * 3/16 = 63/256 + 18/512 = (126+18)/512 = **28%**

(X, Y) : 9/64 + 36/256 + 6/128 + 3/256 = (36+36+12+3)/256 = 87/256 = **33,9%**

(X, X)_Mx_ : 9/256 + 12/256 + 6/128 + 6/2048 + 3/256 = (9+12+3)/256_0,09_ + 6/128_0,046_ + 6/2048_0,003_

= (24*8 + 16*6 + 6)/2048 = 294/2048 = **14,3%**

(X, X)_Mc_ : 6/1024 + 1/1024 + 1/1024 = 8/1024 = **0,78%**

(X, Y)_MC_ : 1/32 * (12/32 + 6/8 + 7/128 + 1/32 ) = 1/32 * 1,21 = **3,78%**

(X, X)_A_ : 6/1024 + 3/1024 = 9/1024 = **0,88%**

(X, Y)_A_ : 36/1024 + 6/256 + 9/4096 + 3/1024= 6/256 + 9/4096 + 39/1024

(4*39 + 16*6 + 9) / 4096 = = **6,37%**

Sex ratio = 6,37/0,88 = **7,24**

**3^rd^ generation**

0,28 * **[** 3/8 (X, X), 1/2 (X, Y), 1/8 (X, X)_MX_ **] _3_**

0,34 * **[** 3/8 (X, X), 3/8 (X, Y), 1/8 (X, X)_MX_, 1/32 (X, Y)_MC_, 3/32 (X, Y)**_A_** **] _1_**

0,14 * **[** 3/16(X, X), 1/4(X, Y), 1/4(X, X)_MX_, 1/32(X, X)_MC_, 1/8(X, Y)_MC_, 1/32(X, X)**_A_**, 1/8(X, Y)**_A_** **] _4_**

0,008 * **[** 3/8 (X, X)_MX_, 1/8 (X, X)_MC_ + 7/32 (X, Y)_MC_ + 9/32 (X, Y)**_A_** **] _5_**

0,04 * **[** 3/8 (X, Y), 3/8 (X, X)_MX_, 1/32 (X, X)_MC_, 1/32 (X, Y)_MC_, 3/32 (X, X)**_A_**, 3/32 (X, Y)**_A_** **] _2_**

**=>** Likelihood of phenotypes:

(X, X) : 0,28*3/8 + 0,34*3/8 + 0,14 *3/16 = **0,26**

(X, Y) : 0,28/2 +0,34*3/8 + 0,14/4 + 0,04*3/8 = **0,33**

(X, X)_MX_ : 0,28/8 + 0,34/8 + 0,28/8 + 3*0,008/8 + 0,12/8 = **0,13**

(X, X)_MC_ : 0,14/32 + 0,008/8 + 0,04/32 = **0,06**

(X, Y)_MC_ : 0,34/32 + 0,14/8+ 0,008*7/32+ 0,04/32 = **0,031**

(X, X)**_A_** = 0,14/32 + 0,04*3/32 = **0,008**

(X, Y)**_A_** = 0,34/32 + 0,14/8 + 0,008*9/32 + 0,04*3/32 = **0,034**

Sex ratio in severe ASD = **4**

including MC : (34 + 18)/ (0,8 + 03) = 3,8

including MX : (34 + 18)/ (0,8 + 03 + 26) = 1,7

No offspring (severe ASD) : 0,008 + 0,034 = **4%**

**4^th^ generation**

0,26 * **[3+] =** 0,26 * **[** 3/8 (X, X), 1/2 (X, Y), 1/8 (X, X)_MX_ **] _3_**

0,33 * **[1+] =** 0,33 * **[** 3/8 (X, X), 3/8 (X, Y), 1/8 (X, X)_MX_, 1/32 (X, Y)_MC_, 3/32 (X, Y)**_A_** **] _1_**

0,13 * **[4+] =** 0,13 * **[** 3/16(X, X), 1/4(X, Y), 1/4(X, X)_MX_, 1/32(X, X)_MC_, 1/8(X, Y)_MC_, 1/32(X, X)**_A_**, 1/8(X, Y)**_A_** **] _4_**

0,06 * **[5+] =** 0,06 * **[** 6/16 (X, X)_MX_, 1/8 (X, X)_MC_ + 7/32 (X, Y)_MC_ + 9/32 (X, Y)**_A_** **] _5_**

0,031 *** [2+] =** 0,031 *** [** 3/8 (X, Y), 3/8 (X, X)_MX_, 1/32 (X, X)_MC_, 1/32 (X, Y)_MC_, 3/32 (X, X)**_A_**, 3/32 (X, Y)**_A_** **] _2_**

**=>**

(X, X) : 0,26*3/8 + 0,33*3/8 + 0,13*3/16 = **0,25**

(X, Y) : 0,26/2 + 0,33*3/8 + 0,13/4 + 0,31*3/8 = **0,4**

(X, X)_MX_ : 0,26/8 + 0,33/8 + 0,13/4 + 0,06*6/16 + 0, 031*3/8 = **0,14**

(X, X)_MC_ : 0,13/32 + 0,06/8 + 0,031/32 = **0,13**

(X, Y)_MC_ : 0,33/32 + 0,13/8 + 0,06*7/32 + 0,031/32 = **0,04**

(X, X)**_A_** : 0,13/32 + 0,031*3/32 = **0,007**

(X, Y)**_A_** : 0,13/8 + 0,06*9/32 + 0,031*3/32 = **0,036**

Sex ratio in severe ASD = **5**

including MC : 0,6

Sum of likelihoods: 0,25 + 0,4 + 0,14 + 0,13 + 0,04 + 0,007 + 0,036 = **100%**
